# The predictive value of machine learning for the response to anti-TNF-α therapy in patients with Crohn’s disease: a systematic review and meta-analysis

**DOI:** 10.3389/fimmu.2026.1891348

**Published:** 2026-09-30

**Authors:** Yuchen Lin, Siyuan Sun, Siyi Yang, Jinhui Sun

**Affiliations:** Department of Gastroenterology, Dongzhimen Hospital of Beijing University of Chinese Medicine, Beijing, China

**Keywords:** Crohn’s disease, machine learning, systematic review, therapy response, TNF-α blockers, anti-TNF-α

## Abstract

**Background:**

This meta-analysis aimed to evaluate the performance of machine learning (ML) in predicting the response to anti-tumor necrosis factor-α (anti-TNF-α) therapy in individuals with Crohn’s disease (CD), providing an evidence-based basis for the construction or update of prediction tools.

**Methods:**

PubMed, Cochrane Library, Embase, and Web of Science were comprehensively searched for studies published up to November 18, 2025 on ML predictive models for the therapeutic response to anti-TNF treatment in individuals with CD. The Prediction model Risk Of Bias Assessment Tool (PROBAST) was used to assess the risk of bias in the eligible articles. Model performance was assessed using the concordance index (C-index), and a meta-analysis was conducted.

**Results:**

A total of 29 studies involving 6,697 patients with CD were included. Of these studies, 22 focused on the response to anti–TNF-α therapy, 6 on mucosal healing, and 1 on other outcomes. The meta-analysis demonstrated that for predicting the treatment response to anti–TNF-α treatment, in the validation dataset, the C-indices were 0.762 for ML models based on clinical features (CFs), 0.852 for models based on radiomic features, and 0.854 for models based on CFs combined with radiomic features. In internal validation, the C-indices for models based on CFs, radiomics, and CFs combined with radiomics were 0.773 (95% CI: 0.711–0.841), 0.840 (95% CI: 0.781–0.904), and 0.830 (95% CI: 0.780–0.883), respectively. In the external validation set, models based on radiomics (0.865, 95% CI: 0.783–0.955) and models based on CFs combined with radiomics (0.883, 95% CI: 0.816–0.955) outperformed models based on CFs (0.733, 95% CI: 0.645–0.833). Furthermore, the C-index of models based on endoscopic mucosal response was 0.794.

**Conclusions:**

hile ML shows promise in predicting the therapeutic effects of anti-TNF-α treatment in individuals with CD, there are some methodological limitations. Future research should integrate multi-center, geographically diverse datasets to enhance the generalizability of models. Improving model robustness will help identify non-responders early, ultimately promoting the development of personalized therapeutic strategies and precision medicine.

**Systematic review registration:**

https://www.crd.york.ac.uk/PROSPERO/, identifier CRD420251276470.

## Introduction

Crohn’s disease (CD) is a persistent inflammatory bowel disease (IBD) that may involve every portion of the digestive tract, predominantly the terminal ileum and colon, leading to intestinal impairment and disability. Inflammation is typically segmental, asymmetrical, and transmural, leading to complications (e.g., strictures, fistulas, or abscesses), for which surgery is often required (1). The prevalence of CD is the highest in Europe (322 cases per 100,000 persons in Germany) and North America (319 cases per 100,000 persons in Canada) (2). Multiple studies have demonstrated that the incidence of CD is stable or declining in North America and Europe. Nevertheless, the incidence has been increasing in newly industrialized regions in Asia, Africa, and South America since 1990 (2). A prospective population-based cohort study in Norway suggests that about half of the patients develop stenosis or penetrating complications within 20 years of diagnosis (3). A meta-analysis based on multiple population-based studies further indicates that, before biologics are widely used, nearly 50% of CD patients require related surgery within 10 years of diagnosis (4). These findings indicate that it is difficult to control the disease with traditional treatment models. However, a recent meta-analysis based on a contemporary cohort population born after 2000 shows that with the widespread use of biologics, the need for surgery in CD patients within 10 years after diagnosis has significantly decreased to 26.2% (5), indicating that refined treatment models have significantly delayed the progression of CD. However, according to the American Gastroenterological Association (AGA) Institute Technical Review (6), 1-year clinical and endoscopic recurrence rates range from 20% to 40% and 30% to 90% respectively, while the cumulative re-surgery rates is 25% at 5 years and 35% at 10 years. The long-term clinical management of this persistent disease is challenging.

Based on the American College of Gastroenterology (ACG) Clinical Guideline in 2025, CD is commonly treated with induction or maintenance therapy, based on the patient’s condition and disease severity. Medical therapies for CD predominantly include supportive care (i.e., diet-based approaches) and corticosteroids and advanced therapies (i.e., anti-TNF-α therapy, anti-integrins, anti-interleukins, and Janus kinase inhibitors) (7). Anti-tumor necrosis factor-α (TNF-α), as a core pro-inflammatory mediator of CD, has become a cornerstone therapy for inducing and maintaining remission in moderate to severe active CD (8). The mechanism of anti-TNF-α therapy in CD is multifaceted. These agents first neutralize soluble tumor necrosis factor (sTNF) while simultaneously antagonizing its transmembrane isoform (tmTNF) to dampen early inflammatory cascades. In addition, anti-TNF-α blocks TNFR2-mediated survival signals transmitted by mucosal CD14+ macrophages, indirectly inducing apoptosis of pathogenic T cells, a response restricted to the inflamed intestinal microenvironment. Furthermore, Fc gamma receptor (FcγR) promotes the polarization of M2 wound-healing macrophages, contributing to the repair of mucosa. Concurrently, epithelial barrier integrity is restored, and pathological mucosal angiogenesis is inhibited, further reducing tissue injury. Collectively, these processes rebalance the intestinal immunomodulatory network, resolving chronic inflammation and reducing the risk of recurrence. Currently, anti-TNF-α drugs for CD approved by FDA/EMA include infliximab (IFX), adalimumab (ADA), and certolizumab (CZP). Although all three drugs target TNF-α, their molecular structures differ fundamentally. IFX is a chimeric IgG1κ monoclonal antibody (25% murine variable region), while ADA is a fully human IgG1 monoclonal antibody, both retaining the complete IgG1 Fc fragment. However, CZP is a human Fab’ fragment modified by PEGylation, naturally lacking the Fc fragment. This structural heterogeneity may explain their different clinical effects. IFX and ADA can simultaneously achieve clinical remission and mucosal healing. It has been reported that IFX maintains a clinical remission rate of 31% up to 54 weeks, while the mucosal healing rate of ADA at 24 weeks is 24%. In contrast, the mucosal healing rate of CZP is only 4%–8%, and its primary non-response (PNR) rate is as high as 71% (IFX: 40%, ADA: 41%) (9, 10). Anti-TNF-α therapy is strongly recommended for managing moderately to severely active CD (7). Anti-TNF-α treatment is efficacious for reducing the use of steroid therapy and may provide more benefits for certain populations, such as steroid-refractory, steroid-intolerant or steroid-dependent patients (11).

Two treatment-failure patterns of anti-TNF therapy are observed in clinical practice. PNR refers to no improvement in clinical signs or symptoms after the induction phase. Secondary non-response or loss of response (SLOR) is defined as an initial response to the therapy, followed by subsequent loss of clinical response (12).

The treatment failure of anti-TNF therapy involves intertwined mechanisms. PNR may be caused by both pharmacokinetic factors (increased drug clearance, either immune-mediated or non-immune-mediated) and pharmacodynamic (mechanistic) factors (8, 10). Baseline factors for PNR include the lack of target antigens, different structures of agents (e.g., no Fc segment in certolizumab pegol), and specific disease phenotypes (13–16). In contrast, SLOR is mainly attributable to acquired factors, including the production of anti-drug antibodies, variability in pharmacokinetics, and changes in pharmacodynamic pathways (10, 13, 14). The mechanisms of PNR and SLOR are partially overlapping. The activation of IL-23/Th17 axis is observed in patients with no response to anti-TNF therapy and may play a significant role in both PNR and SLOR, where TNFR2+IL23R+ T cells increase and IL-23p19 and IL-17A are upregulated (14). In clinical practice, SLOR patients typically show a higher response rate to dose optimization than PNR patients, which indirectly indicates a greater role of reversible pharmacokinetic factors in SLOR (9).

Multiple meta-analyses have suggested that anti-TNF-α agents are the core treatment drugs for moderate to severe CD. However, their deep remission rate decreases significantly with longer treatment duration, with significant heterogeneity (17–19). Approximately 25% of patients do not respond to anti-TNF treatment, while one-third of initial responders lose response by the end of the first year (20). Traditionally, patients are monitored, and medications are switched only after anti-TNF-α therapy becomes ineffective, which is inappropriate. A meta-analysis of 18 studies by Gisbert et al. indicates that non-response to first-line anti-TNF therapy is an independent risk factor for decreased response to subsequent biologics. If high-risk individuals who do not respond to anti-TNF therapy are not identified early and their medication is not tailored accordingly, the subsequent biologic response rate will be lower (21). Furthermore, early optimized treatment for high-risk individuals with SLOR can avoid unnecessary changes in medical prescriptions (22). Early identification of PK and PD types can also avoid blindly increasing drug doses for PD patients and prematurely changing drugs for PK patients (23). Furthermore, persistent inflammation during the progression of CD increases the risks of fibrosis, stenosis, and surgery. If reactive treatment intensification is not used until clinical recurrence is observed, transmural fibrosis may have already occurred. Early identification of SLOR can prevent new stenosis, perforation, abdominal abscess, and surgery due to delays in the window period. Additionally, anti-TNF-α treatment is expensive. Identifying patients unresponsive to anti-TNF therapy can avoid wasting medication costs and reduce the cost of remedial treatment after recurrence. Therefore, early identification of high-risk groups for PNR or SLOR is significant and economical. Clinically, it is difficult for physicians to specifically determine whether a patient is at high risk. Commonly used clinical methods, such as trough concentration and anti-drug antibody testing, only have moderate predictive value and are insufficient (24). There are no early prediction tools for the response to anti-TNF therapy in clinical practice. Machine learning (ML) has advantages in processing high-dimensional heterogeneous data.

ML refers to a research method to develop intricate data models based on specific data features or images, aiming to explore the relationship of biomarkers with outcome events, or to perform diagnosis, classification, or prediction of outcomes. In addition to traditional clinical features (CFs), radiomics features can also be used to develop common ML models. Radiomics, as a novel and continuously evolving high-throughput quantitative technique, is gradually becoming a key component of radiogenomics and computational gastroenterology. Modeling based on radiomics features is to use manual or semi-automatic segmentation (including deep learning) to extract and summarize radiomics features from images to construct models for disease diagnosis, treatment response, and prognostic assessment. Unlike traditional qualitative visual assessment, this technique can mine thousands of features from standardized cross-sectional images (such as CTE/MRE), covering multi-dimensional parameters such as texture, morphology, and signal intensity, thus providing a new, non-invasive method for understanding the potential heterogeneity and pathophysiological microenvironment of CD. Radiomics has been increasingly explored in recent years due to its ability to non-invasively capture the pathological heterogeneity of transmural lesions in CD. CD is caused by a multi-network pathogenic mechanism and may progress into fibrosis and activation of mesenteric fat. CD lesions not only affect the entire intestinal wall but also spread to the perimesenchyme. Thus, the pathological characteristics of CD are different from those of ulcerative colitis. These interconnected pathological mechanisms can be quantitatively assessed by imaging features, such as layered enhancement and wall edema (immunoinflammatory), T2 low signal with delayed enhancement (fibrosis), comb-like signs and creeping fat (mesenteric fat remodeling), and sinus tracts/abscesses (penetrating behavior). Therefore, radiomics can translate complex pathophysiological processes into quantifiable imaging features by capturing multi-network pathological changes, the histological microenvironment, and macroscopic imaging findings. Accordingly, a biologically meaningful system of imaging biomarkers has been established, providing a promising research direction for CD (25). ML has been increasingly employed in the clinical management of CD. Grassi et al. have systematically reviewed the application of ML in processing medical images of CD, and preliminarily suggested that it is feasible to extract and use radiomics features of CD to develop predictive models (26).

Meta-analysis can provide insights into predicting the response to anti-TNF-α treatment, and can screen risk factors and evaluate efficacy. Pauwels et al. have summarized 14 studies from 11 countries with 1317 patients to conduct an individual participant data (IPD) meta-analysis. They have optimized the predictive model for the recurrence of CD after the discontinuation of anti-TNF treatment. The calibration benchmarks reported in their study provide evidence-based references for ML in the risk stratification of recurrence (27). Ten Bokkel Huinink et al. further conducted a meta-analysis based on 12 articles involving 309 patients. They found that patients with perianal fistula CD with quiescent intestinal lesions may maintain remission for 2 years after discontinuing anti-TNF treatment. This finding provides an evidence-based basis for the discontinuation of anti-TNF treatment, offering research directions for predicting efficacy in subdivided populations (28). Furthermore, meta-analyses on the predictive accuracy of biomarkers can provide a reference for variable selection and threshold setting in subsequent predictive models by summarizing the predictive efficacy of individual factors. For example, a meta-analysis conducted by Freeman et al. (24) based on 31 studies indicates that the overall performance of existing predictors of the efficacy of anti-TNF-α therapy (such as drug trough concentration and anti-drug antibodies) is limited. Developing more accurate predictive models is necessary. Although recent systematic reviews have reviewed the application of ML in predicting treatment response in inflammatory bowel disease, existing evidence is derived from mixed cohorts of CD and ulcerative colitis (29). Furthermore, the number of existing models is small, and no quantitative meta-analysis specifically for anti-TNF treatment has been performed. Research that systematically integrates raw data and quantifies the predictive efficacy of ML for the response to anti-TNF treatment in CD is still lacking. Further research is needed to explore the true performance of ML in predicting the responses to anti-TNF treatment in CD patients.

In summary, although ML has been preliminarily used to predict the response to anti-TNF therapy in CD, existing evidence is fragmented, and research quality varies. There is no quantitative meta-analysis to systematically evaluate its predictive performance. Furthermore, the impact of different data types and modeling strategies on predictive accuracy remains unclear. Therefore, this meta-analysis aimed to summarize the predictive performance of ML for the response to anti-TNF-α therapy in CD. This study would provide a reference for the iterative improvement of risk assessment tools for adverse prognoses and offer more objective evidence for the use of anti-TNF therapy. Simultaneously, it is expected to optimize the allocation of medical resources, reduce unnecessary overtreatment or undertreatment that leads to disease progression, provide a reference for the development of precise intervention strategies for high-risk groups, and offer an evidence-based foundation for individualized anti-TNF treatment in CD.

## Methods

### Study registration

This study was performed in accordance with the Transparent Reporting of Multivariable Prediction Models for Individual Prognosis or Diagnosis–Systematic Reviews and Meta-Analyses (TRIPOD-SRMA) guideline (30) and was retrospectively registered in PROSPERO (ID: CRD420251276470, https://www.crd.york.ac.uk/PROSPERO/view/CRD420251276470).

### Eligibility criteria

#### Inclusion criteria

P (Population): CD patients diagnosed based on clinical presentation, endoscopy, imaging, and pathology, aged ≥18 years, receiving anti-TNF-α monotherapy or in combination with immunosuppressants (including infliximab, adalimumab, and certolizumab).

I (Intervention): Specific modeling algorithms included, but are not limited to, logistic regression, Cox proportional hazards model, LASSO regression, elastic net, random forest, gradient boosting tree (XGBoost/LightGBM/CatBoost), and support vector machine (SVM). Models were classified into three types based on modeling features as follows:

1. Models based on clinical risk factors: These models only included routinely available CFs, encompassing demographic characteristics (age and sex), baseline disease characteristics (disease duration, Montreal classification, lesion location, history of bowel resection/perforation), laboratory indicators (baseline CRP, fecal calprotectin, albumin levels), and medication history (history of glucocorticoid/immunosuppressant exposure).
2. Models based on radiomics features: These models were constructed based solely on high-throughput quantitative features extracted from medical images.
3. Combined models based on both radiomic and CFs: These models integrated both clinical risk factors and radiomics features.

C (Comparison): The predictive performance of models based on different features was compared, providing references for supporting model selection in clinical practice.

O (Outcome): Response to anti-TNF-α treatment, including clinical response (including PNR and SLOR), and mucosal healing.

T (Timing): Predictors should be collected before or at the start of anti-TNF-α treatment, and the outcome at a preset time point after treatment was used as the predictive target. Models in which features were dynamically updated during follow-up were not included.

S (Setting): Real-world observational cohort studies (including hospital cohorts, multi-center registry prospective or retrospective cohorts), RCTs, and their secondary analyses.

#### Exclusion criteria

The following studies were excluded:

1. Publication type: meeting abstracts;
2. Study scope: analyses limited to risk factors or correlational relationships without developing predictive models;
3. Data availability: studies in which prediction models were developed, but discrimination and calibration metrics were unavailable.

### Data sources and search strategy

We systematically searched four major databases, encompassing Cochrane Library, Embase, PubMed, and Web of Science, up to November 18, 2025. The search strategy was designed by combining subject headings (e.g., MeSH, Emtree) with free-text terms. The search strategy for databases is described in **Supplementary Table S1**. Data sources and search strategies were collaboratively developed by two investigators. Specifically, Y.C. Lin drafted the initial search strings, which were independently reviewed and refined by J.H. Sun.

### Study selection

The searched publications were imported into EndNote. After removing duplicates, the titles and abstracts of the remaining reports were screened to exclude ineligible reports. The full texts of potentially eligible articles were then retrieved to identify eligible studies. Study selection was performed independently by two investigators (S-Y. Yang and Y.C. Lin) and subsequently cross-checked. Any disagreements were settled by a third investigator (J.H. Sun).

### Data extraction

Before data extraction, a standard spreadsheet was developed. Extracted data included article title, DOI, first author, country, study design, patient source, agent, treatment protocol, definition of treatment response, follow-up duration, number of events, total sample size, number of positive events in the model, total events in the model, derivation method of validation dataset, overfitting handling approach, missing data management strategy, variable/feature selection technique, types of models implemented, predictor variables, and model performance metrics.

Data extraction was performed independently by two investigators (S.Y. Sun and Y.C. Lin) and subsequently cross-checked. Any disagreements were resolved by a third investigator (J.H. Sun).

### Risk of bias in studies

The Prediction model Risk Of Bias Assessment Tool (PROBAST) (31) was used to assess the risk of bias in four domains: participants, predictors, outcome, and analysis. Each domain included several signaling questions, which were answered with “yes”, “probably yes”, “probably no”, “no”, or “no information”. A domain was judged as low risk of bias only if all signaling questions in the domain were answered with “yes” or “probably yes”. A domain was judged as high risk of bias if one or more signaling questions were answered with “no” or “probably no” and the related shortcoming was considered relevant to the systematic review question and likely to seriously reduce the model’s predictive performance. Otherwise, the domain was judged as unclear. The risk of bias was independently assessed by two investigators (S.Y. Yang and Y.C. Lin) and then cross-checked. Any disagreements were resolved by a third investigator (J.H. Sun).

### Synthesis methods

The meta-analysis of c-index was performed to evaluate the accuracy of ML models. For studies that did not report the 95% confidence intervals (CIs) or standard errors (SEs) of the c-index, we derived standard errors based on the method established by Debray et al. (32). Due to the clinical and methodological heterogeneity across studies, a random-effects model was adopted to account for between-study heterogeneity in the meta-analysis of c-index. Furthermore, a bivariate mixed-effects model was initially planned to pool sensitivity and specificity. However, due to incomplete reporting of classification metrics across included studies, the meta-analyses of sensitivity and specificity were not performed. The data analyses were performed using R version 4.5.2 (R Development Core Team, Vienna, Austria. URL: http://www.R-project.org). Statistical analyses were performed independently by two investigators (S.Y. Sun and Y.C. Lin) and subsequently cross-checked. Any disagreements were resolved by a third investigator (J.H. Sun).

## Results

### Study selection

Initially, 4,796 records were searched from databases. After 2,100 duplicates were removed, the titles and abstracts of the remaining studies (n=2696) were read to eliminate 2,658 irrelevant studies. The full texts of 38 studies were comprehensively evaluated. Then, 9 articles were excluded due to unpublished conference abstracts (n = 4), failure to predict the response to anti-TNF-α treatment (n = 3), and investigating risk factors without developing a predictive model (n = 2). Ultimately, 29 articles were included in this review (**Figure 1**) (33–61).

**Figure 1:**
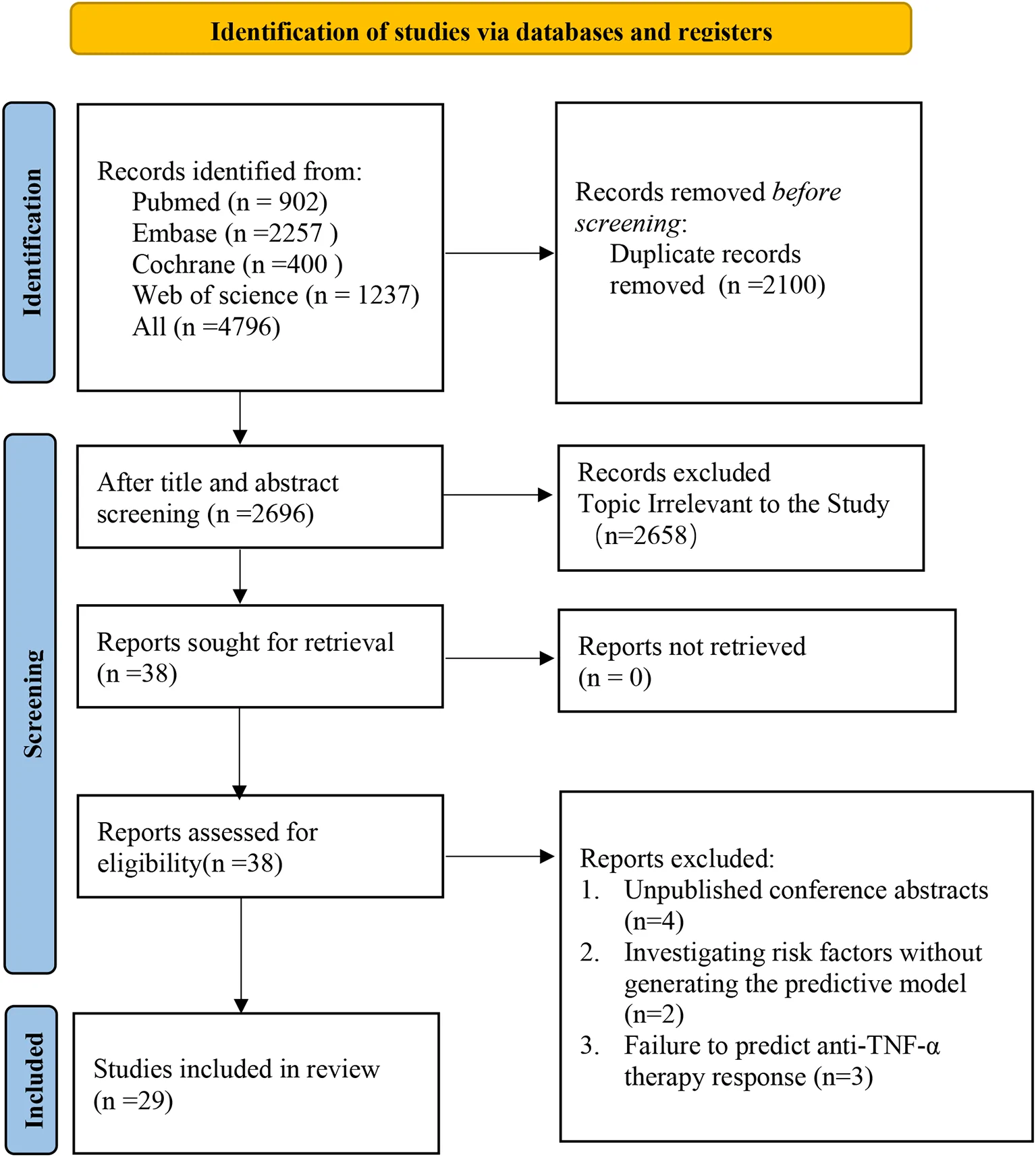
PRISMA flow diagram for literature screening.

### Study characteristics

The 29 eligible articles were predominantly published between 2015 and 2025. These studies were conducted across four countries, and most studies were from China. Regarding study design, there were 3 prospective cohort studies and 26 retrospective cohort studies. Regarding data sources and study settings, 13 articles were multicenter, while the remaining studies were single-center. The medications included infliximab (n=23), adalimumab (n=2), and certolizumab (n=1). All studies explicitly defined therapeutic response. Specifically, 22 studies focused on the response to anti-TNF-α therapy; 6 studies focused on predicting post-treatment mucosal healing; another study predicted anti-drug antibody (ADA) positivity. A total of 21 studies explicitly reported the complete follow-up duration, ranging from 12 to 104 weeks, involving 6697 patients with CD. The random split validation was used in 13 studies, cross-validation in 2 studies, bootstrap resampling in 2 studies, external validation in 3 studies, random split combined with cross-validation in 1 study, internal random split combined with external validation in 7 studies, and phased validation in 2 studies. Ten studies extracted both radiomic features and CFs, although their final models varied: some studies retained only CFs, some used exclusively radiomics, and others combined both features. The remaining studies used CFs (n=14) or radiomics alone (n=5) (**Table 1**; **Supplementary Table S2**).

**Table 1:** The features of the eligible articles.

No.	First author	Publication year	Country	Study design	Patient source	Medication	Predicted outcome	Follow-up time	Total sample size	Missing-Data exclusion rate (%)	Model category	Features
1	Pianpian Xia	2025	China	Retrospective Cohort Study	Single-Center	Adalimumab	Treatment Response	52w	244	0	RF, GBM, XGBoost, GBM, CatBoost, AdaBoost	Clinical Features
2	Yiting Wang	2025	China	Retrospective Cohort Study	Single-Center	Infliximab	Anti-drug Antibody Positivity	42w	606	19.41	RF, KNN, XGBoost, GBM, LR, SVM, DT	Clinical Features
3	Y. Wang	2025	China	Retrospective Cohort Study	Single-Center	Anti-TNF therapy	Treatment Response	52w	105	Not Mentioned	DL	Radiomic Features
4	Fang Wang	2025	China	Retrospective Cohort Study	Single-Center	Adalimumab	Treatment Response	12w	68	38.73	LR	Clinical Features
5	Yun Qiu	2025	China	Retrospective Cohort Study	Multicenter	Infliximab	Clinical Relapse	104w	746	11.51	XGBoost, LR, LASSO	Clinical Features
6	Jingjing Liu	2025	China	Retrospective Cohort Study	Single-Center	Infliximab	Treatment Response	26w	85	0	LR	Clinical Features
7	Xi Li	2025	China	Retrospective Cohort Study	Single-Center	Infliximab	Treatment Response	54w	161	45.24	LR	Combined Radiomic and Clinical Features
8	Shuai Li	2025	China	Retrospective Cohort Study	Multicenter	Infliximab	Treatment Response	54w	220	26.17	LR	Combined Radiomic and Clinical Features
9	Lixuan Li	2025	China	Retrospective Cohort Study	Single-Center	Infliximab	Post-Treatment Mucosal Healing	30w	179	Not Mentioned	LR	Clinical Features
10	Zhuoyan Chen	2025	China	Retrospective Cohort Study	Multicenter	Infliximab	Treatment Response	52w	134	55.03	XGBoost, AdaBoost, KNN, NB, RF, BR, SVM	Radiomic Features
11	Tian Yang	2024	China	Retrospective Cohort Study	Single-Center	Infliximab	Treatment Response	54w	184	Not Mentioned	LR	Combined Radiomic and Clinical Features
12	Yangdi Wang	2024	China	Retrospective Cohort Study	Multicenter	Infliximab	Post-Treatment Mucosal Healing	14w	231	13.16	SVM	Radiomic Features
13	Chao-Tao Tang	2024	China	Retrospective Cohort Study	Multicenter	Infliximab	Treatment Response	14w	175	20.45	LR	Combined Radiomic and Clinical Features
14	Fulong Song	2024	China	Retrospective Cohort Study	Single-Center	Infliximab	Treatment Response	52w	137	18.45	LR	Combined Radiomic and Clinical Features
15	Liang Chen	2024	China	Prospective Cohort Study	Multicenter	Anti-TNF therapy	Post-Treatment Mucosal Healing	14w	443	Not Mentioned	LR	Clinical Features
16	Weimin Cai	2024	China	Retrospective Cohort Study	Multicenter	Infliximab	Treatment Response	54w	263	7.72	SVM	Combined Radiomic and Clinical Features
17	Chao Zhu	2023	China	Retrospective Cohort Study	Multicenter	Infliximab	Treatment Response	54w	181	7.65	LR	Combined Radiomic and Clinical Features
18	Chen Yueying	2023	China	Retrospective Cohort Study	Single-Center	Infliximab	Treatment Response	52w	188	10.48	LR	Radiomic Features
19	Rosario Medina-Medina	2023	Spain	Prospective Cohort Study	Multicenter	anti-TNF therapy	Treatment Response	52w	113	Not Mentioned	LR	Clinical Features
20	Chao Zhu	2022	China	Retrospective Cohort Study	Multicenter	Infliximab	Post-Treatment Mucosal Healing	26w	106	34.97	LR	Combined Radiomic and Clinical Features
21	Xuehua Li	2022	China	Retrospective Cohort Study	Multicenter	Infliximab	Post-Treatment Mucosal Healing	14w	244	12.54	LR	Combined Radiomic and Clinical Features
22	Pavine L.C. Lefevre	2022	United States	Secondary Analysis of RCT Data	Multicenter	Certolizumab Pegol	Treatment Response	26w	964	W6:8;W26:11	LR	Clinical Features
23	Jing Feng	2022	China	Retrospective Cohort Study	Single-Center	Infliximab	Treatment Response	54w	322	3.3	LR	Combined Radiomic and Clinical Features
24	Chen Yueying	2021	China	Retrospective Cohort Study	Single-Center	Infliximab	Treatment Response	54w	136	33.66	LR	Clinical Features
25	Xiao-Qi Ye	2021	China	Retrospective Cohort Study	Multicenter	Infliximab	Post-Treatment Mucosal Healing	14w	343	Not Mentioned	LR	Clinical Features
26	Yong Li	2021	China	Retrospective Cohort Study	Single-Center	Infliximab	Treatment Response	14w	174	9.84	RF	Clinical Features
27	Li Li	2021	China	Prospective Cohort Study	Single-Center	Infliximab	Treatment Response	14w	260	2.26	RF	Clinical Features
28	Yueying Chen	2021	China	Retrospective Cohort Study	Single-Center	Infliximab	Treatment Response	54w	186	26.48	LR	Radiomic Features
29	Thomas Billiet	2015	Beligum	Retrospective Cohort Study	Single-Center	Infliximab	Treatment Response	14w	201	Not Mentioned	LR	Clinical Features

RF, Random forest; GBM, Gradient boosting machine; XGBoost, eXtreme gradient boosting; GBM, Gradient boosting machine; CatBoost, Categorical boosting; AdaBoost, Adaptive boosting; LR, Logistic regression; LASSO, Least Absolute Shrinkage and Selection Operator; KNN, k-Nearest neighbors; NB, Naive Bayes; BR, Bridge regression; DL, Deep learning.

### Risk of bias in studies

Of the 29 included studies, 28 were cohort studies, and one was a secondary analysis of data from an RCT. Thus, the risk of bias in the participant domain was low. Regarding the domain of predictors, predictor assessments were consistent across all participants and were evaluated without knowledge of the outcome data. In some original studies, variables with more than 25% missing data were considered difficult to impute and were therefore considered invalid. Data sparsity usually referred to some categorical variables with a percentage below 5%, or continuous variables with extremely abnormal data, and these abnormal data were not adequately processed in original studies. The eligible studies did not have severe missing data or sparsity. Consequently, the predictor domain was rated as low risk. Regarding the outcome domain, the classification and definition of outcomes were appropriate. We did not find any excessive data sparsity in the current definition of categorical variables, or any violations of international clinical guidelines (62), conference consensus (63), and indicator definitions (64–66) in the process of defining categorical variables. Outcomes were determined consistently for all participants without knowing predictor information, and the time interval between predictor assessment and outcome determination was reasonable. Thus, this domain was evaluated as low risk. In the analysis domain, 26 of the 29 included studies were at high risk of bias, 1 study at low risk, and 2 studies at unclear risk (**Figure 2**). Among the 26 high-risk studies, 8 were at high risk solely because of an events per variable (EPV) ratio <10, no independent validation dataset, or a validation dataset with fewer than 100 cases. Thirteen studies used complete-case analysis (deletion) for missing data and had EPV <10 or validation issues, resulting in a high risk of bias. The remaining 5 studies were at high risk solely due to complete-case analysis. Because the two studies with unclear risk did not specify the number of radiomic or genetic features, the EPV and validation issues mentioned above could not be resolved.

**Figure 2:**
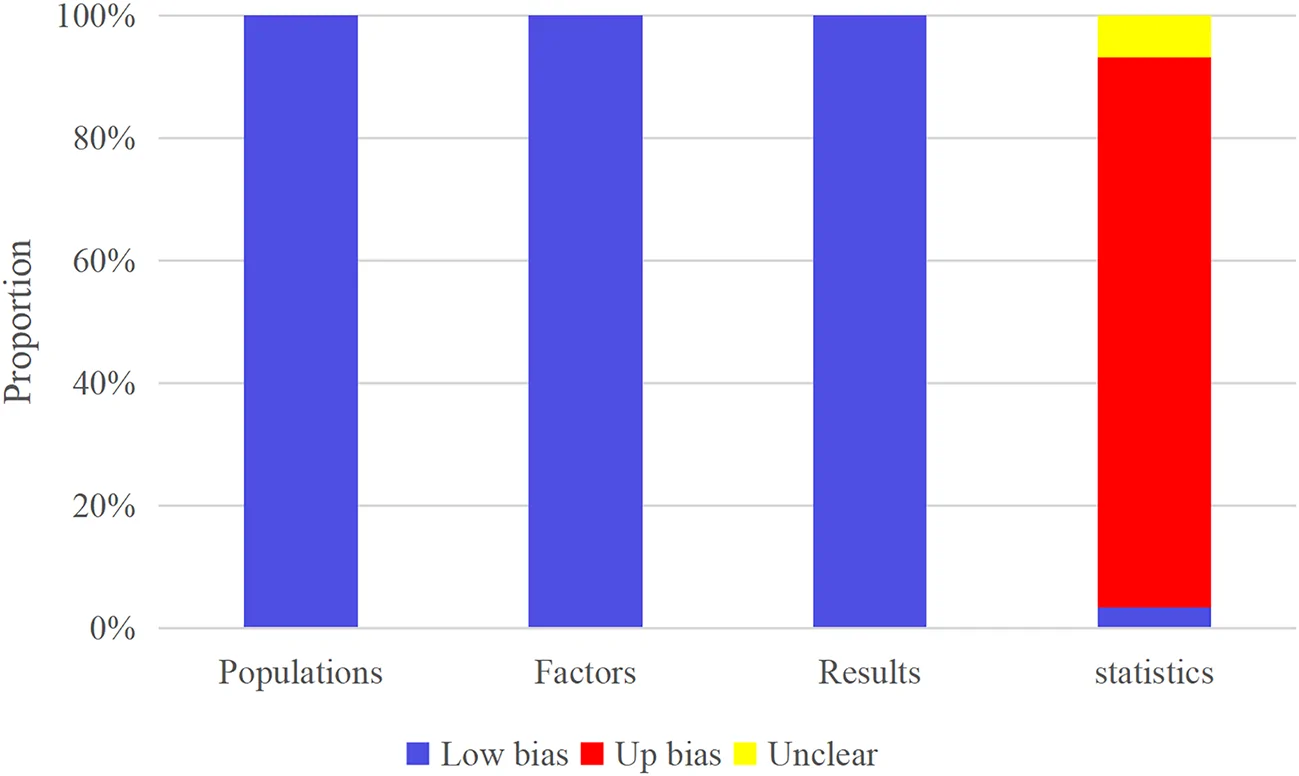
Risk of bias assessment (PROBAST).

### Meta-analysis of treatment responses

#### Prediction models based on clinical features

A total of 17 studies developed models based on CFs to predict responses to anti-TNF treatment. For the 14 models in the training dataset, the C-index was 0.818 (95% CI: 0.772–0.867). LR was the most frequently used algorithm, with a C-index of 0.798 (95% CI: 0.754–0.845) (**Supplementary Figure S1**; **Table 2**). In the subgroup analysis by medications, there were 12 models for predicting response to infliximab, with a pooled C-index of 0.814 (95% CI: 0.761–0.872) (**Table 2**).

**Table 2:** Meta-analysis of C-indices for clinical feature-based machine learning models in predicting treatment response to anti-TNF-α therapy in Crohn’s disease.

Clinical features	Training set			Validation set		
	n	C-index (95%CI)	Tau2	n	C-index (95%CI)	Tau2
Model						
XGBoost	1	0.959 (0.928-0.991)		2	0.685 (0.601-0.781)	0
RF				2	0.923 (0.877-0.972)	0
LR	12	0.798 (0.754-0.845)	0.007	14	0.748 (0.689-0.813)	0.014
SVM	1	0.910 (0.861-0.962)	NA	1	0.710 (0.620-0.813)	NA
Medication						
Adalimumab	1	0.866 (0.780-0.961)	NA	2	0.912 (0.852-0.975)	0.001
Infliximab	12	0.814 (0.761-0.872)	0.011	14	0.730 (0.673-0.792)	0.012
Certolizumab	1	0.800 (0.766-0.836)	NA	2	0.710 (0.655-0.769)	0
Anti-TNF-α therapy				1	0.919 (0.863-0.978)	NA
Response						
Primary Non-Response	6	0.831 (0.782-0.884)	0.003	10	0.819 (0.749-0.896)	0.015
Secondary Loss of response	6	0.805 (0.720-0.900)	0.017	7	0.682 (0.627-0.743)	0
Treatment Failure	2	0.818 (0.652-1.00)	0.024	2	0.700 (0.619-0.791)	0
Overall	14	0.818 (0.772-0.867)	0.009	19	0.762 (0.710-0.818)	0.015

Treatment failure encompasses primary non-response (PNR) and secondary loss of response (SLOR).

XGBoost, extreme gradient boosting; RF, random forest; LR, logistic regression; SVM, support vector machine.

In the validation dataset (n=20 models), the overall C-index was 0.762 (95% CI: 0.710–0.818). LR was the most frequently used algorithm (n=14), with a C-index of 0.748 (95% CI: 0.689–0.813). Subgroup analysis by medications revealed that 14 models were developed to predict the response to infliximab, with a pooled C-index of 0.730 (95% CI: 0.673–0.792). The C-indexes for the models in predicting PNR (n=10), SLOR (n=7), and treatment failure were 0.819 (95% CI: 0.749–0.896), 0.682 (95% CI: 0.627–0.743), and 0.700 (95% CI: 0.619–0.791), respectively (**Supplementary Figure S2**; **Table 2**).

In the internal validation dataset, the overall pooled C-index of 14 models was 0.773 (95% CI: 0.711–0.841). There were three model types. LR (n=12 models) was the most frequently used algorithm, with a pooled C-index of 0.740 (95% CI: 0.674–0.813) (**Supplementary Figure S3**). In the subgroup analysis by medications, 10 models were developed for predicting the treatment response to infliximab, with a pooled C-index of 0.725 (95% CI: 0.657–0.800).

For the external validation dataset, the pooled C-index of five models was 0.733 (95% CI: 0.645–0.833) (**Supplementary Figure S4**). According to the subgroup analysis by medications, most studies focused on infliximab, and the C-index was 0.733 (95% CI: 0.672–0.800). The C-indexes of the models in predicting PNR, SLOR and treatment failure were 0.820 (95%CI: 0.606–1.00), 0.657 (95%CI: 0.572-0.756) and 0.710 (95%CI: 0.620-0.813), respectively (**Supplementary Table S3**).

#### Prediction models based on radiomic features

A total of 11 studies developed models based on radiomic features to predict the response to anti-TNF treatment. In the training dataset (comprising 11 models across 4 model types), the overall C-index was 0.854 (95% CI: 0.824–0.886). LR was the predominant algorithm (n=8), with a pooled C-index of 0.866 (95% CI: 0.831–0.902) (**Supplementary Figure S5**). According to the subgroup analysis by medications, most models were designed to predict the response to infliximab (n=10), with a pooled C-index of 0.855 (95% CI: 0.822–0.888).

In the validation dataset (including 12 models across 4 algorithm types), the pooled C-index was 0.852 (95% CI: 0.807–0.900). LR was used for 9 models (C-index: 0.873, 95% CI: 0.829–0.920). XGBoost showed the best performance, with a pooled C-index of 0.910 (95% CI: 0.847–0.977) (**Supplementary Figure S6**). Notably, the pooled C-index of all evaluated model types was higher than 0.70. According to the subgroup analysis by medications, 11 models were developed to predict the response to infliximab (C-index: 0.857, 95% CI: 0.812–0.906). The pooled C-indexes for predicting PNR, SLOR, and treatment failure were 0.934 (95% CI: 0.886–0.895), 0.835 (95% CI: 0.774–0.901), and 0.822 (95% CI: 0.745–0.908), respectively (**Table 3**).

**Table 3:** Meta-Analysis of C-indices for radiomic feature-based machine learning models in predicting the treatment response to anti-TNF-α treatment in Crohn’s disease.

Radiomic features	Training set			Validation set		
	n	C-index (95%CI)	Tau2	n	C-index (95%CI)	Tau2
Model						
XGBoost	1	0.843 (0.780-0.911)	NA	1	0.910 (0.847-0.977)	NA
ViT	1	0.843 (0.756-0.941)	NA	1	0.745 (0.590-0.941)	NA
LR	8	0.866 (0.831-0.902)	0.002	9	0.873 (0.829-0.920)	0.002
SVM	1	0.780 (0.717-0.848)	NA	1	0.720 (0.630-0.823)	NA
Medication						
Infliximab	10	0.855 (0.822-0.888)	0.002	11	0.857 (0.812-0.906)	0.005
Anti-TNF therapy	1	0.843 (0.756-0.941)	NA	1	0.745 (0.590-0.941)	NA
Response						
Primary Non-Response	1	0.909 (0.842-0.981)	NA	2	0.934 (0.886-0.985)	<0.001
Secondary Loss of response	5	0.859 (0.809-0.912)	0.003	5	0.835 (0.774-0.901)	0.002
Treatment Failure	5	0.836 (0.800-0.873)	0.001	5	0.822 (0.745-0.908)	0.008
Overall	11	0.854 (0.824-0.886)	0.002	12	0.852 (0.807-0.900)	0.005

Treatment failure encompasses primary non-response (PNR) and secondary loss of response (SLOR).

XGBoost: extreme gradient boosting; ViT: vision transformer; LR: logistic regression; SVM: support vector machine.

In the subgroup analysis by validation approach, the internal validation dataset included 8 models (2 types), with an overall pooled C-index of 0.840 (95% CI: 0.781–0.904). LR remained the most frequently used algorithm (n=7; C-index 0.849, 95% CI: 0.788–0.915) (**Supplementary Figure S7**). Most internal models were designed to predict the response to infliximab (n=7; pooled C-index 0.849, 95% CI: 0.788–0.915). Meanwhile, the external validation dataset comprised 4 models (3 types). All these models were designed to predict the response to infliximab, with a pooled C-index of 0.865 (95% CI: 0.783–0.955)(**Supplementary Figure S8**). The C-indexes for models in predicting PNR, SLOR, and treatment failure were 0.902 (95% CI: 0.833–0.977), 0.909 (95% CI: 0.815–1.00), and 0.815 (95% CI: 0.648–1.00), respectively (**Supplementary Table S4**).

#### Prediction models based on combined radiomic and clinical features

Seven studies developed ML models based on CFs combined with radiomics to predict the response to anti-TNF-α therapy, all exclusively focusing on infliximab. In the training dataset, there were 7 models (comprising LR and SVM), and the C-index was 0.869 (95% CI: 0.818–0.923). LR was the predominant algorithm (n=6), with a C-index of 0.861 (95% CI: 0.803–0.924)(**Supplementary Figure S9**).

In the validation dataset (including 8 models [LR and SVM]), the pooled C-index was 0.854 (95% CI: 0.816–0.893). LR remained the most frequently used algorithm (n=7), with a C-index of 0.852 (95% CI: 0.804–0.902) (**Supplementary Figure S10**). The pooled C-indexes for predicting SLOR and treatment failure were 0.852 (95% CI: 0.801–0.906) and 0.847 (95% CI: 0.781–0.919), respectively (**Table 4**).

**Table 4:** Meta-analysis of C-indices for machine learning models integrating clinical and radiomic features to predict the treatment response to anti-TNF-α treatment in Crohn’s disease.

Combined radiomic and clinical features	Training set			Validation set		
	n	C-index (95%CI)	Tau2	n	C-index (95%CI)	Tau2
Model						
LR	6	0.861 (0.803-0.924)	0.007	7	0.852 (0.804-0.902)	0.001
SVM	1	0.910 (0.866-0.956)	NA	1	0.850 (0.778-0.929)	NA
Medication						
Infliximab	7	0.869 (0.818-0.923)	0.006	8	0.854 (0.816-0.893)	0
Response						
Secondary Loss of response	5	0.857 (0.787-0.934)	0.008	6	0.852 (0.801-0.906)	0.001
Treatment Failure	2	0.901 (0.864-0.941)	0	2	0.847 (0.781-0.919)	0
Overall	7	0.869 (0.818-0.923)	0.006	8	0.854 (0.816-0.893)	0

Treatment failure encompasses primary non-response (PNR) and secondary loss of response (SLOR).

logistic regression; SVM: support vector machine.

Subgroup analysis revealed that in the internal validation dataset, there were 6 LR models, with a pooled C-index of 0.830 (95% CI: 0.780–0.883). Meanwhile, in the external validation dataset, the pooled C-index was 0.883 (95% CI: 0.816–0.955) (**Supplementary Figure S11**,**12**). The pooled C-indexes for predicting SLOR and treatment failure were 0.921 (95% CI: 0.836–1.00) and 0.850 (95% CI: 0.778–0.929), respectively (**Supplementary Table S5**).

### Meta-regression

We performed meta-regressions on the training and validation sets based on follow-up time, proportion of positive events, medication type, model type, variable type, source of validation set, and risk of bias. No significant heterogeneity due to these factors was found in the training set. However, in the validation set, medication type and variable type appeared to introduce significant heterogeneity. Due to the limited number of studies, the results need to be carefully interpreted (**Table 5**, **6**).

**Table 5:** Meta-regression of machine learning-predicted Anti-TNF-α treatment response in the training cohort.

Factors	Coefficient	SE	Z	p	95%CI
Follow Time(month)	-0.0001	0.0009	-0.1570	0.875	-0.0020 ~ 0.0017
Proportion	-0.0005	0.0012	-0.4451	0.656	-0.0029 ~ 0.0018
Medication					
Infliximab(Ref)					
Others	-0.0030	0.0547	-0.0541	0.957	-0.1102 ~ 0.1042
Model					
LR(Ref)					
Others	0.0544	0.0455	1.1962	0.231	-0.0347 ~ 0.1435
Variable type					
Clinical featrues(Ref)					
Radiomics features	0.0286	0.0325	0.8776	0.380	-0.0352 ~ 0.0924
Clinical + Radiomic features	0.0510	0.0376	1.3562	0.175	-0.0227 ~ 0.1247
Risk of bias					
High bias(Ref)					
Others	-0.0086	0.0566	-0.1514	0.880	-0.1195 ~ 0.1023

Proportion: Proportion of positive events.

**Table 6:** Meta-regression of machine learning-predicted anti-TNF-α treatment response in the validation cohort.

Factors	Coefficient	SE	Z	p	95%CI
Follow Time(month)	-0.0013	0.0010	-1.3986	0.162	-0.0032 ~ 0.0005
Proportion	-0.0007	0.0015	-0.4358	0.663	-0.0037 ~ 0.0023
Medication					
Infliximab(Ref)					
Others	0.1422	0.0524	2.7150	0.007	0.0395 ~ 0.2449
Model					
LR(Ref)					
Others	0.0182	0.0451	0.4039	0.686	-0.0702 ~ 0.1066
Source of validation cohort					
Inner validation					
External validation	0.0402	0.0389	1.0334	0.301	-0.0360 ~ 0.1164
Variable type					
Clinical features (Ref)					
Radiomics features	0.0817	0.0356	2.2984	0.022	0.0120 ~ 0.1514
Clinical + Radiomic features	0.1135	0.0422	2.6870	0.007	0.0307 ~ 0.1962
Risk of bias					
High bias(Ref)					
Others	0.0711	0.0653	1.0891	0.276	-0.0569 ~ 0.1992

Proportion: Proportion of positive events.

### Publication bias

In the training and validation sets for predicting treatment response, funnel plots suggested a significant publication bias among studies (**Supplementary Figure S13**, **S14**), with Egger’s test P < 0.05. However, in the prediction of mucosal healing, no significant publication bias was observed in either the training or validation sets (**Supplementary Figure S15**, **S16**), with Egger’s test P > 0.05.

### Meta-analysis of post-treatment mucosal healing

A total of six studies constructed models for predicting the endoscopic mucosal response to anti-TNF-α treatment in patients with CD. In the training dataset, the overall C-index was 0.834 (95% CI: 0.806-0.863). Subgroup analysis by feature type indicated that the pooled C-indices were 0.800 (95% CI: 0.752-0.851) for CF-based models (n=6), 0.857 (95% CI: 0.817-0.900) for radiomics-based models, and 0.872 (95% CI: 0.826-0.921) for models based on CFs combined with radiomics (**Supplementary Figure S17**). According to the subgroup analysis by medications, most models (n=10) were designed to predict the treatment response to infliximab, with a C-index of 0.833 (95% CI: 0.802-0.866).

In the validation dataset (comprising 15 models), the overall C-index was 0.794 (95% CI: 0.765-0.825). There were 7 models based on CFs (C-index: 0.756, 95% CI: 0.708-0.808), 5 models based on radiomics (C-index: 0.815, 95% CI: 0.767-0.867), and 3 models based on CFs combined with radiomics (C-index: 0.820, 95% CI: 0.757-0.888) (**Supplementary Figure S18**). Similarly, most models (n=14) were designed to predict the response to infliximab, with a pooled C-index of 0.817 (95% CI: 0.715-0.934) (**Table 7**).

**Table 7:** Meta-analysis of C-indices for machine learning models for predicting post-treatment mucosal healing in Crohn’s disease following anti-TNF-α treatment.

Post-treatment mucosal healing	Training set			Validation set		
	n	C-index (95%CI)	Tau2	n	C-index (95%CI)	Tau2
Variable						
Clinical Features	6	0.800 (0.752-0.851)	0.004	7	0.756 (0.708-0.808)	0.001
Radiomics Features	3	0.857 (0.817-0.900)	0	5	0.815 (0.767-0.867)	0
Clinical + Radiomic Features	2	0.872 (0.826-0.921)	0	3	0.820 (0.757-0.888)	0
Medication						
Adalimumab	1	0.828 (0.759-0.903)	NA	1	0.817 (0.715-0.934)	NA
Infliximab	10	0.833 (0.802-0.866)	0.002	14	0.792 (0.761-0.824)	<0.001
Overall	11	0.834 (0.806-0.863)	0.001	15	0.794 (0.765-0.825)	<0.001

### Review of studies on predicting other outcomes of anti-TNF-α therapy

One study on predicting anti-drug antibody (ADA) positivity following anti-TNF therapy developed seven internally validated models. Among these models, XGBoost was the optimal predictive model, with a C-index of 0.899(95% CI: 0.841–0.957). The C-indices for K-nearest neighbors (KNN) and GBM were 0.766(95% CI: 0.804–0.958) and 0.888 (95% CI: 0.824–0.953), respectively.

## Discussion

### Summary of the main findings

This review included 29 articles to assess the performance of ML models for predicting the response to anti-TNF therapy. Notably, ML models demonstrated promising performance in both internal and external validation datasets. The models were developed primarily based on CFs, radiomic features, or their combination. Compared to traditional clinical indicators, radiomics features appear to have greater potential in predicting the response to anti-TNF-α treatment. In the internal validation set, the C-index of radiomics-based models was 0.840, which was similar to that of the combined model (C-index: 0.830), indicating no significant improvement. However, in the external validation set, the combined model (C-index: 0.883) outperformed the radiomics-based model (C-index: 0.865). This difference between internal and external validation results suggests that the observed superiority of the combined model in external validation should be interpreted with caution. Given the relatively limited sample size in the external validation set, further studies with larger cohorts are still needed to verify whether the addition of CFs provides additional gains to the model. Moreover, given the inherent risk of bias in the statistical analysis of the included studies, the lack of effective external validation, and the limited reporting of calibration, we should not be overly optimistic about the current findings.

### Comparison with previous reviews

Previous literature has investigated the application of ML in predicting pharmacological treatment responses in CD. For instance, Ganesh et al. (67) have conducted a narrative review to discuss factors associated with monoclonal antibody therapy in CD and reviewed the current applications of ML for predicting treatment responses. Similarly, Nguyen et al. (68) have summarized ML-based diagnostic and prognostic models in inflammatory bowel disease, including 13 studies with 30,219 patients (including four multicenter studies, three of which were international, and eight single-center studies). Their results reveal that ML models outperform traditional statistical approaches in risk prediction, despite a high risk of bias in most models. However, these previous reviews predominantly focus on reviewing the application of ML models but do not quantitatively synthesize their predictive performance. Hence, the actual predictive performance of ML in this field remains inconclusive.

Therapeutic responses are clearly defined in most studies, typically quantified by the CD Activity Index (CDAI), Harvey Bradshaw Index (HBI), Patient-Reported Outcome 2 (PRO2), Simple Endoscopic Score for CD (SES-CD), Crohn’s Disease Endoscopic Index of Severity (CDEIS), C-reactive protein (CRP), or fecal calprotectin (FC). CDAI is an 8-item weighted composite, incorporating 7-day stool frequency, abdominal pain, general well-being, extraintestinal complications, antidiarrheal use, abdominal mass, hematocrit, and percentage deviation from standard weight. Clinical remission is conventionally defined as a CDAI total score < 150 or a reduction ≥100 points from the pretreatment baseline (69). HBI serves as an unweighted simplified tool of CDAI, comprising general well-being, abdominal pain, liquid stools per day, abdominal mass, and extraintestinal complications. Remission is conventionally defined as HBI < 5 (64). The Patient-Reported Outcome 2 (PRO2) for CD is derived from two weighted diary items extracted from the CDAI—specifically, 7-day abdominal pain and stool frequency—to reflect patient-related disease activity. Clinical response is conventionally defined as **≥** 50% decrease in PRO2 (63, 70). SES-CD evaluates four morphological variables, including ulcer size, ulcerated surface area, proportion of surface affected, and presence of narrowing across the ileum and four colonic segments. Endoscopic response is conventionally considered as a ≥50% reduction from the baseline (65). CDEIS comprises six weighted variables: the proportion of segments with deep ulcerations and superficial ulcerations, the mean percentage of surface involved by disease, the mean percentage of surface occupied by ulceration, and fixed scores for non-ulcerated stenosis and ulcerated stenosis, assessed across five intestinal segments (rectum, left colon, transverse colon, right colon, and ileum). Endoscopic healing per STRIDE-II is defined as CDEIS ≤ 3 with no ulcerations (63, 66). CRP and FC are utilized as serological and stool-based biomarkers of intestinal inflammation, respectively. The two indicators serve as intermediate targets in treat-to-target strategies, and are conventionally standardized as CRP ≤ upper limit of normal and FC 100–250 μg/g (63).

Among the 29 included studies, the definitions for the efficacy of TNF therapy in CD were not standardized, leading to heterogeneity. This heterogeneity is largely attributable to inconsistent methodologies across ML studies, such as bias and flawed validation practices reported in systematic reviews, rather than limitations of the algorithm (71, 72). Less than half of the included studies utilized objective tools such as SES-CD or CDEIS to determine the treatment efficacy. The remaining studies relied heavily on clinical symptom scores (e.g., CDAI) or combined validation indicators (CPR or FC) to assess treatment response, or adverse prognostic events and physician assessments. This resulted in significant variations in positive event rates. Furthermore, none of the included studies used clinical, endoscopic, and biochemical composite outcomes as positive events for model training, which is recommended by STRIDE-II (63). Most studies focused only on a single dimension of efficacy. Accordingly, the clinical translation of research results is challenging. Clinicians often struggle to assess the reliability of predictive tools developed based on different standards. Furthermore, with different definitions of outcomes, cross-sectional comparisons of the incremental cost-effectiveness of different models are infeasible, reducing the reliability of model performance in clinical practice. Future research should reduce heterogeneity in definitions by standardizing the definition of the efficacy of anti-TNF treatment. Outcome definitions should adhere to STRIDE-II standards, covering three core dimensions: clinical, endoscopic, and biochemical domains. Additionally, follow-up duration should be standardized. Furthermore, outcome assessments should be conducted blindly by a third party to avoid potential biases caused by physicians’ subjective judgement.

We also performed meta-regression to explore additional sources of heterogeneity. No significant heterogeneity due to these factors was observed in the training set. In contrast, variable type and medication type may be the sources of heterogeneity in the validation set. Specifically, compared to models based on CFs, models based on radiomics features and those combining clinical and radiomics features showed higher C-index, at approximately 0.082 (P = 0.022) and 0.114 (P = 0.007), respectively. While this significant difference implies that incorporating radiomics features might enhance predictive performance to some extent, these findings should be interpreted with caution. Radiomics enables us to extract high-throughput quantitative features from medical images. Theoretically, these features may help capture heterogeneity in lesions imperceptible to the naked eye, which may more comprehensively reflect the complex pathophysiological mechanism of intestinal inflammation in CD. However, whether this association is entirely attributable to differences in feature types needs to be further validated in more independent cohorts.

Meanwhile, the results of subgroup analysis by medication type also reached statistical significance (P = 0.007). The analysis showed that the C-index of models for other anti-TNF-α agents was approximately 0.142 higher than those for infliximab. This finding suggests that the performance of predictive models may differ to some extent among various anti-TNF-α drugs. We speculate that this discrepancy might be related to differences in molecular structure, immunogenicity, or pharmacokinetic profiles among these agents, where subtle pharmacological variations could lead to heterogeneity in clinical responses. Additionally, existing studies mostly focus on infliximab, while sample sizes for other drugs remain relatively limited. This imbalance may also affect the stability of model performance to some extent. Therefore, it should be cautious to use current models for predicting the response to different anti-TNF-α agents. Future high-quality studies encompassing a broader range of drugs are needed to further validate this finding and elucidate the underlying clinical mechanisms.

Feature selection remains challenging. Conventional predictive variables generally include CFs, image-derived radiomics features, and genomic profiles. Our study primarily focused on clinical and radiomics features. Radiomics has been increasingly used in recent years. This method allows us to extract vast quantitative data from medical images, offering a non-invasive means to evaluate heterogeneity within lesions. Consequently, radiomics serves as a pivotal tool for diagnosis, prognosis, and biopsy targeting of diseases, and plays a pivotal role in developing personalized treatment plans and monitoring therapeutic efficacy (73, 74). Conversely, while radiomics has garnered substantial attention in clinical practice, there are several challenges. For instance, there is heterogeneity in imaging across diverse scanner vendors and acquisition protocols. The impact of this heterogeneity on the robustness of models is still unclear, and the generalizability of models without rigorous preprocessing in external validation datasets remains concerning (75). Moreover, region-of-interest (ROI) segmentation relies heavily on operators’ expertise (76). Multi-center research and clinical translation are subject to irreproducibility and misinterpretation due to no consensus guidelines for validating and reporting AI-induced modifications to image statistics, quantification, and textural heterogeneity (77). Although standardized radiological reporting templates (e.g., RadReport) and standardized lexicons (e.g., RadLex) have been developed recently, there is still substantial variability across radiologists and institutions. Reports are written using largely semi-structured, narrative free texts. This unstructured text can lead to discrepancies between radiologists and referring clinicians in the interpretation of these texts (78). Furthermore, the inherent black box nature of radiomics is more apparent in small-sample studies. In small, single-center studies, technical homogeneity is high, and thus it is easier for models to capture technical covariates, potentially leading to spurious features. For example, if a study includes CD patients who are all underweight and receive low-flow-rate contrast agent injections, resulting in specific features on imaging, the model might mistakenly identify this feature as a meaningful biomarker, affecting its robustness and clinical applicability. CFs are inherently interpretable because each variable corresponds to a defined clinical entity. Accordingly, CF-based models can reflect established clinical risk factors for adverse outcomes in CD (35). CFs encompass biological behaviors that radiomics cannot capture, such as disease duration, age, presence of complications, and previous medication and surgical history. For instance, a CD patient with an anal fistula or a history of biologic injections who experiences clinical recurrence may have a relatively poor prognosis. CFs can reflect these risk factors in models, filling a gap in radiomics in this area. Additionally, CFs are less dependent on equipment parameters and may be more stable than radiomics. To some extent, CFs can compensate for the heterogeneity of radiomics. In summary, CFs and radiomics may provide complementary information, and the combined model based on both CFs and radiomics may be more efficient. Nonetheless, further large-scale external validations are still needed to confirm their added value.

Although radiomics can non-invasively quantify heterogeneity in lesions to monitor treatment response, it has the aforementioned limitations. Future research should combine radiomic features with CFs like disease duration and prior treatment history to develop models. Furthermore, imaging acquisition protocols should be standardized, and research should be reported according to TRIPOD. With these standardized procedures and rigorous validation, radiomics may become a reliable tool for real-time clinical decision-making.

While ML models for predicting the responses to anti-TNF-α treatment in CD have been developed and show promising performance, there are several significant methodological limitations in the included studies, hindering their clinical translation. In some included studies, EPV was insufficient, possibly due to the epidemiological characteristics of CD. Even in high-prevalence regions like Europe and the Americas, the annual number of anti-TNF-α-naïve patients enrolled in a single center is extremely limited. Based on the service population, disease activity rate, and proportion of indications for medication, about 50 to 100 cases are observed at a single tertiary IBD center annually. This number is similar to data from Billiet et al. (58), who enrolled 201 patients over 14 years, with an average annual enrollment rate of approximately 14%. Furthermore, long-term follow-up is needed for assessing the response to anti-TNF-α treatment, further reducing the effective sample size. Therefore, the insufficiency of EPV is not an isolated phenomenon, but a structural barrier to building high-precision prediction models for CD globally. The insufficiency of EPV can induce model overfitting, leading to biased AUC values and artificially inflating the model’s real-world performance. This model could result in erroneous decisions and delays in treatment in clinical practice. For example, if a candidate for first-line IFX treatment is misclassified as a primary non-responder by a radiomics-based model with low EPV, clinicians might select more expensive ustekinumab, even though the patient actually tolerates IFX well. This not only incurs a huge health economic burden but also unnecessarily exposes the patient to the adverse effects of potent immunosuppressants. Conversely, misclassifying a non-responder as a responder delays the opportunity to escalate treatment, accelerates disease progression to the fibrosis phenotype, and ultimately leads to the need for surgical intervention. Additionally, the studies included in our review have geographical bias. Most participants in the studies were from China, and only a small portion of participants were from other countries. Hence, the study population is nonrepresentative of patients in many regions with fastest-growing CD cases, such as Africa, South Asia, Latin America, and the Middle East. These populations have different genetic structures, gut microbiota characteristics, and environmental exposures. Hence, the generalizability of the constructed predictive models to global regions is limited.

Therefore, large-scale, multi-center research covering five major geographical regions (East Asia, South Asia, Africa, Latin America, and Europe and the Americas) is needed to break down data silos and build robust models with statistical power. Additionally, standardized public datasets should be established, relying on organizations such as the International Organization for Inflammatory Bowel Diseases (IOIBD) and the European Crohn’s and Colitis Organization (ECCO). Technically, federated learning (FL) offers a promising privacy-preserving computational path to jointly traincross-center models without exchanging raw data, which can not only ensure data sovereignty but also increase the sample size. Furthermore, improving the methodology of small-sample studies is also crucial. For example, introducing Bayesian models with embedded prior information and using evidence from historical cohorts or previous literature to estimate parameters can effectively buffer the statistical uncertainty caused by insufficient EPV. Simultaneously, given the specific factors of certain geographical environments in practical applications (such as parasitic infection in Africa, different dietary patterns in various regions, and religious customs), more suitable models should be developed based on specific application scenarios.

Additionally, the high risk of bias in some articles was attributable to the use of complete-case analysis to handle missing data. Many studies on predicting the response to anti-TNF-α are conducted by clinical gastroenterologists rather than methodological experts. They may believe that only extractable data are true, and imputing missing values may be considered data manipulation. They may not have realized that excluding cases with missing values could lead to bias. Additionally, journals often prioritize model performance over the handling method of missing values, and some journals often restrict the length of the method section. Therefore, the handling methods for missing values in this field are often neglected. In the complete-case analysis, some community patients and patients with mild symptoms are excluded, and some core data are neglected, leading to potential bias. Future research should strictly adhere to the TRIPOD guidelines (79), employ appropriate methods to handle missing values (such as multiple imputation), and report the missing value rate and reasons for each variable, the methods used to handle missing values, and perform sensitivity analyses.

This meta-analysis analyzed the performance of ML models predicting the response to anti-TNF-α therapy in CD. However, these findings should be interpreted cautiously, given the underlying methodological limitations. The risk of bias in the analysis domain was high in 26 included studies, primarily due to insufficient EPV, absence of independent validation, or use of complete-case analysis. Insufficient EPV can induce overfitting, leading to optimistic C-index values in training sets but reduced generalizability in independent cohorts. This overestimation is likely amplified in studies with limited sample sizes. Moreover, in complete-case analysis for missing data, patients with milder symptoms or incomplete follow-up may be excluded, which may introduce selection bias. Accordingly, the resulting models are not applicable to real-world populations, ultimately reducing their clinical utility (reflected by the C-index). These biases do not exist in isolation but intersect with the epidemiologic features of CD. For instance, a single center can only enroll a limited number of participants annually, and further sample attrition may occur during long-term follow-up. All these factors hinder the development of predictive models. Therefore, the reported C-index should be interpreted in light of these potential limitations.

Furthermore, a significant risk of publication bias was observed in our study, particularly in the prediction of treatment responses. In both the training and validation sets, the funnel plots (**Supplementary Figure S13**, **S14**) were significantly asymmetrical, and results of Egger’s test were positive (P<0.05). Studies on high-performance ML models are more likely to be published, whereas studies reporting moderate C-index or AUC values are likely to be rejected by journals. Hence, the performance of existing models may be overstated. In contrast, the funnel plots for predicting mucosal healing were relatively symmetrical (**Supplementary Figure S15**, **S16**), and Egger’s test showed no statistical significance (P>0.05). This difference may reflect that predicting mucosal healing is still in a relatively early exploratory stage. The included studies on mucosal healing were fewer, and many were exploratory analyses. Nevertheless, small studies with null or subtle effects may be rejected for publication. Therefore, we should fully consider the potential overestimation of model performance caused by publication bias when interpreting the results, especially regarding the predictive power for treatment response. Future research should be reported in accordance with the TRIPOD guidelines, and it is encouraged to publish zero or negative results to gradually correct this systemic bias.

Moreover, the selection of the optimal model remains challenging. Research often focuses on constructing and comparing multiple ML models (e.g., LR and decision trees) (80). Among the included studies in our review, 4 studies developed multiple ML models to ultimately identify the best-performing model. Nevertheless, this method has inherent limitations. First, model interpretability varies significantly. Traditional algorithms, such as LR, decision trees, linear discriminant analysis, Cox regression, and actuarial risk models, are inherently interpretable. They can directly quantify associations between predictors and clinical endpoints or event risks, thereby improving the precision of clinical judgments. Conversely, complex ML models typically have a black-box nature. Even though contemporary techniques have been used to improve their interpretability, the translation of ML models into real-world clinical practice is still challenging (81).

The 29 studies included in our review did not adequately report sensitivity, specificity, and precision (PPV and NPV). Therefore, a meta-analysis of these indicators is infeasible. Only few studies reported all three indicators, and the reporting was not standardized. Most studies only reported the optimal indicator from the training set, and did not report the indicators in the validation set. Furthermore, some studies failed to clearly explain the criteria for selecting the thresholds for the classification indicators (e.g., the Youden index and clinical decision threshold) and only reported the indicators at the optimal threshold. This is because threshold segmentation is difficult in some studies due to the low EPV. Future research should report sensitivity, specificity, PPV and NPV in the training set, internal validation set, and external validation set, rather than only using AUC as the performance metric. Moreover, the criteria for selecting the thresholds for classification metrics (e.g., Youden index, clinical net benefit maximization threshold, and preset clinical threshold) should also be explained, and the ranges of sensitivity and specificity under different thresholds should be reported. Alternatively, tools should be provided for physicians to customize thresholds for clinical application. For outcomes with low event rates, sensitivity or specificity under high thresholds should be reported to avoid missing or misclassifying cases in different scenarios.

Existing research generally does not quantitatively assess calibration, and the reporting of calibration curves and decision curve analysis (DCA) is not standardized. Among the 29 included studies, only 69% (20/29) reported calibration results; 55% (16/29) conducted DCA; and only 34% (10/29) simultaneously conducted multi-cohort validation, reported quantitative calibration indicators, and clearly elucidated threshold ranges of DCA. The remaining studies all had varying degrees of reporting limitations. In some studies, methods and results were disconnected. For example, the studies by Cai et al. (45) and Tang et al. (42) only provided calibration and DCA figures in the result section but failed to explicitly mention the two indicators in the method section, thus not complying with TRIPOD reporting standards. The threshold setting of DCA was not aligned with clinical practice in some studies. For example, Chen Yueying et al. (57) set the threshold range to 5%–95% in their study, far exceeding the reasonable range of 10%–50% for changing medication for IFX. Such a large range has no practical clinical significance. Some studies confused the concepts of Hosmer-Lemeshow test (HL) and calibration curve. For instance, Feng et al. (52) reported the p-value of HL but did not provide a calibration curve, making it impossible to determine the specific deviations in the low-predictivity or high-probability intervals. Moreover, most studies only vaguely stated that the model had a net benefit when interpreting DCA results, without clearly elucidating specific interventions. For example, Song et al. (43) only reported the DCA results for three models to show the difference in net benefit but did not explain that specific measures such as medication switching, dosage increase or combined immunosuppression should be taken when the predicted risk exceeds X%. Hence, it is difficult for clinicians to use these findings in clinical settings. In clinical practice, poorly calibrated models can lead to biased risk estimation. For example, a model might predict a 40% risk of PNR, but the actual risk is only 10%, resulting in overtreatment; or a model might predict 20%, but the risk actually reaches 60%, potentially missing the optimal time to switch medications. Models lacking DCA cannot provide guidance on the timing of intervention in clinical practice. For example, if an ML model with high AUC is uncalibrated and lacks DCA, doctors cannot determine the reliability of the model or whether to switch medications when the prediction is >20%. Ultimately, they can only rely on experience rather than the model to make treatment decisions, hindering the application of ML in clinical practice.

To address the aforementioned issues, future studies on developing prediction models for the response to anti-TNF-α therapy in CD patients must strictly adhere to methodological standards and TRIPOD/TRIPOD-ML reporting guidelines, clearly elucidate the calibration curve (including HL test, mean absolute calibration error, and calibration slope/intercept) and DCA in the method section. After ML models output predicted values, Platt scaling or isotonic regression calibration must be performed to avoid probability bias. Both visual calibration plots and quantitative indicators should be provided. Reasonable threshold ranges for DCA should be set, avoiding meaningless wide ranges. It is recommended to provide clinical impact curves (CIC) to show the absolute number of beneficiaries at different thresholds, enhancing clinical readability. Furthermore, specific clinical pathways related to DCA should be specified. For instance, it is recommended to state that if the predicted risk of PNR is >20%, we recommend switching to vedelizumab instead of increasing the IFX dosage. Furthermore, net benefits should be translated into hard endpoints such as reduced surgery rates and lower per capita healthcare expenditure. Besides, single-center studies can divide data into three cohorts: training set, internal validation set, and external test set. Multi-center studies must validate the stability of calibration and DCA in at least two independent centers. When independent external data are unavailable, at least time-split validation should be used to avoid the overfitting of cross-validation. Studies with low event rates should clearly describe the limitations of small sample sizes.

For the clinical translation of predictive models, rigorous validation is necessary. Among our included studies, 10 studies performed external validation. Our review revealed that these ML models consistently showed promising performance across external datasets. Thereby, ML appears to be a viable and robust strategy for predicting therapeutic responses to anti-TNF therapy. Nevertheless, external validation is not universally performed to confirm the generalizability of models across studies, primarily due to data silos. Thus, the findings from these externally validated models must be interpreted with caution.

### Strengths and limitations

This is the first systematic review to evaluate the performance of ML models in predicting the response to anti-TNF-α therapy in individuals with CD. Nevertheless, several limitations must be acknowledged. First, certain model metrics were reported in only a limited number of articles. Consequently, we only performed a qualitative narrative review rather than a quantitative meta-analysis of these metrics. Second, potential publication bias was observed, particularly in studies on treatment response. This bias may contribute to the overestimation of model performance. Third, many original studies did not provide sufficient model performance metrics, such as calibration and clinically applicable probability thresholds. Consequently, given no robust thresholds and limited use of DCA, the actual net clinical benefit of the models in real-world practice remains elusive. Fourth, some subgroup analyses were performed based on a very limited number of studies. The results should be interpreted with caution. Additional studies are needed to further validate these findings.

## Conclusion

ML models show promising performance in predicting the effectiveness of anti-TNF-α treatment in CD. However, current models still have non-negligible methodological limitations, such as insufficient sample sizes, a lack of effective external validation and calibration, and publication bias. Future studies should incorporate multicenter cohorts from geographically diverse regions to develop highly generalizable ML models. Such tools will help early identify CD patients with suboptimal responses to anti-TNF-α therapy, thereby promoting the development of tailored treatment strategies and ultimately realizing the goal of precision medicine.

## Data Availability

The original contributions presented in the study are included in the article/**Supplementary Material**. Further inquiries can be directed to the corresponding author.
