# Supplementary material for: The predictive value of machine learning for the response to anti-TNF-α therapy in patients with Crohn’s disease: a systematic review and meta-analysis
Source: Front Immunol. 2026 Sep 30;17:1891348. doi: 10.3389/fimmu.2026.1891348 (PMC13662062; doi:10.3389/fimmu.2026.1891348)
Supplement: Supplementary file 1 [file Table1.docx]

**Supplementary Materials**

**Supplementary Table S1** Literature search strategy

1.Pubmed

| Search number | Query | Results |
| --- | --- | --- |
| #1 | "Crohn Disease"[Mesh] | 48102 |
| #2 | ((((((Crohn Disease[Title/Abstract]) OR (Crohn's Disease[Title/Abstract])) OR (Crohns Disease[Title/Abstract])) OR (Crohn's Enteritis[Title/Abstract])) OR (Terminal Ileitis[Title/Abstract])) OR (Morbus Crohn[Title/Abstract])) OR (Ileocolitis[Title/Abstract]) | 63638 |
| #3 | ("Crohn Disease"[Mesh]) OR (((((((Crohn Disease[Title/Abstract]) OR (Crohn's Disease[Title/Abstract])) OR (Crohns Disease[Title/Abstract])) OR (Crohn's Enteritis[Title/Abstract])) OR (Terminal Ileitis[Title/Abstract])) OR (Morbus Crohn[Title/Abstract])) OR (Ileocolitis[Title/Abstract])) | 73167 |
| #4 | Machine Learning[MeSH Terms] | 107493 |
| #5 | ((((((((((((((((((((((((((((((((((((((((Machine Learning[Title/Abstract]) OR (artificial intelligence[Title/Abstract])) OR (Transfer Learning[Title/Abstract])) OR (prediction model[Title/Abstract])) OR (risk model[Title/Abstract])) OR (risk score[Title/Abstract])) OR (deep learning[Title/Abstract])) OR (deep networks[Title/Abstract])) OR (deep network[Title/Abstract])) OR (Ensemble Learning[Title/Abstract])) OR (random forest[Title/Abstract])) OR (neural network[Title/Abstract])) OR (neural networks[Title/Abstract])) OR (K-Nearest Neighbor[Title/Abstract])) OR (Support vector machine[Title/Abstract])) OR (SVM[Title/Abstract])) OR (Gradient Boosting Machine[Title/Abstract])) OR (Nomogram[Title/Abstract])) OR (XGBoost[Title/Abstract])) OR (Adaboost[Title/Abstract])) OR (LightGBM[Title/Abstract])) OR (CatBoost[Title/Abstract])) OR (Gradient Boosting[Title/Abstract])) OR (Decision tree[Title/Abstract])) OR (Regression Trees[Title/Abstract])) OR (Naive Bayesian[Title/Abstract])) OR (Multilayer perceptron[Title/Abstract])) OR (Bayesian network[Title/Abstract])) OR (CNN[Title/Abstract])) OR (RNN[Title/Abstract])) OR (LSTM[Title/Abstract])) OR (Generative Adversarial Network[Title/Abstract])) OR (YOLO[Title/Abstract])) OR (MobileNet[Title/Abstract])) OR (ResNet[Title/Abstract])) OR (AlexNet[Title/Abstract])) OR (VGGNet[Title/Abstract])) OR (GoogLeNet[Title/Abstract])) OR (Unet[Title/Abstract])) OR (Radiomics[Title/Abstract])) OR (Radiomic[Title/Abstract]) | 532543 |
| #6 | (Machine Learning[MeSH Terms]) OR (((((((((((((((((((((((((((((((((((((((((Machine Learning[Title/Abstract]) OR (artificial intelligence[Title/Abstract])) OR (Transfer Learning[Title/Abstract])) OR (prediction model[Title/Abstract])) OR (risk model[Title/Abstract])) OR (risk score[Title/Abstract])) OR (deep learning[Title/Abstract])) OR (deep networks[Title/Abstract])) OR (deep network[Title/Abstract])) OR (Ensemble Learning[Title/Abstract])) OR (random forest[Title/Abstract])) OR (neural network[Title/Abstract])) OR (neural networks[Title/Abstract])) OR (K-Nearest Neighbor[Title/Abstract])) OR (Support vector machine[Title/Abstract])) OR (SVM[Title/Abstract])) OR (Gradient Boosting Machine[Title/Abstract])) OR (Nomogram[Title/Abstract])) OR (XGBoost[Title/Abstract])) OR (Adaboost[Title/Abstract])) OR (LightGBM[Title/Abstract])) OR (CatBoost[Title/Abstract])) OR (Gradient Boosting[Title/Abstract])) OR (Decision tree[Title/Abstract])) OR (Regression Trees[Title/Abstract])) OR (Naive Bayesian[Title/Abstract])) OR (Multilayer perceptron[Title/Abstract])) OR (Bayesian network[Title/Abstract])) OR (CNN[Title/Abstract])) OR (RNN[Title/Abstract])) OR (LSTM[Title/Abstract])) OR (Generative Adversarial Network[Title/Abstract])) OR (YOLO[Title/Abstract])) OR (MobileNet[Title/Abstract])) OR (ResNet[Title/Abstract])) OR (AlexNet[Title/Abstract])) OR (VGGNet[Title/Abstract])) OR (GoogLeNet[Title/Abstract])) OR (Unet[Title/Abstract])) OR (Radiomics[Title/Abstract])) OR (Radiomic[Title/Abstract])) | 540,014 |
| #7 | ("Crohn Disease"[Mesh]) OR (((((((Crohn Disease[Title/Abstract]) OR (Crohn's Disease[Title/Abstract])) OR (Crohns Disease[Title/Abstract])) OR (Crohn's Enteritis[Title/Abstract])) OR (Terminal Ileitis[Title/Abstract])) OR (Morbus Crohn[Title/Abstract])) OR (Ileocolitis[Title/Abstract])) AND ((Machine Learning[MeSH Terms]) OR (((((((((((((((((((((((((((((((((((((((((Machine Learning[Title/Abstract]) OR (artificial intelligence[Title/Abstract])) OR (Transfer Learning[Title/Abstract])) OR (prediction model[Title/Abstract])) OR (risk model[Title/Abstract])) OR (risk score[Title/Abstract])) OR (deep learning[Title/Abstract])) OR (deep networks[Title/Abstract])) OR (deep network[Title/Abstract])) OR (Ensemble Learning[Title/Abstract])) OR (random forest[Title/Abstract])) OR (neural network[Title/Abstract])) OR (neural networks[Title/Abstract])) OR (K-Nearest Neighbor[Title/Abstract])) OR (Support vector machine[Title/Abstract])) OR (SVM[Title/Abstract])) OR (Gradient Boosting Machine[Title/Abstract])) OR (Nomogram[Title/Abstract])) OR (XGBoost[Title/Abstract])) OR (Adaboost[Title/Abstract])) OR (LightGBM[Title/Abstract])) OR (CatBoost[Title/Abstract])) OR (Gradient Boosting[Title/Abstract])) OR (Decision tree[Title/Abstract])) OR (Regression Trees[Title/Abstract])) OR (Naive Bayesian[Title/Abstract])) OR (Multilayer perceptron[Title/Abstract])) OR (Bayesian network[Title/Abstract])) OR (CNN[Title/Abstract])) OR (RNN[Title/Abstract])) OR (LSTM[Title/Abstract])) OR (Generative Adversarial Network[Title/Abstract])) OR (YOLO[Title/Abstract])) OR (MobileNet[Title/Abstract])) OR (ResNet[Title/Abstract])) OR (AlexNet[Title/Abstract])) OR (VGGNet[Title/Abstract])) OR (GoogLeNet[Title/Abstract])) OR (Unet[Title/Abstract])) OR (Radiomics[Title/Abstract])) OR (Radiomic[Title/Abstract]))) | 902 |

2.Cochrane

| Search number | Query | Results |
| --- | --- | --- |
| #1 | MeSH descriptor: [Crohn Disease] explode all trees | 2394 |
| #2 | (Crohn Disease):ti,ab,kw OR (Crohn's Disease):ti,ab,kw OR (Crohns disease):ti,ab,kw OR (Crohn's Enteritis):ti,ab,kw OR (Terminal Ileitis):ti,ab,kw | 6722 |
| #3 | (Morbus Crohn):ti,ab,kw OR (Ileocolitis):ti,ab,kw | 78 |
| #4 | #1 or #2 or #3 | 6727 |
| #5 | MeSH descriptor: [Machine Learning] explode all trees | 1222 |
| #6 | (Machine Learning):ti,ab,kw OR (artificial intelligence):ti,ab,kw OR (Transfer Learning):ti,ab,kw OR (prediction model):ti,ab,kw OR (risk model):ti,ab,kw | 45188 |
| #7 | (risk score):ti,ab,kw OR (deep learning):ti,ab,kw OR (deep networks):ti,ab,kw OR (deep network):ti,ab,kw OR (Ensemble Learning):ti,ab,kw | 48388 |
| #8 | (random forest):ti,ab,kw OR (neural network):ti,ab,kw OR (neural networks):ti,ab,kw OR (K-Nearest Neighbor):ti,ab,kw OR (Support vector machine):ti,ab,kw 5545 | 5545 |
| #9 | (SVM):ti,ab,kw OR (Gradient Boosting Machine):ti,ab,kw OR (Nomogram):ti,ab,kw OR (XGBoost):ti,ab,kw OR (Adaboost):ti,ab,kw | 2553 |
| #10 | (LightGBM):ti,ab,kw OR (CatBoost):ti,ab,kw OR (Gradient Boosting):ti,ab,kw OR (Decision tree):ti,ab,kw OR (Regression Trees):ti,ab,kw | 1430 |
| #11 | (Naive Bayesian):ti,ab,kw OR (Multilayer perceptron):ti,ab,kw OR (Bayesian network):ti,ab,kw OR (CNN):ti,ab,kw OR (RNN):ti,ab,kw | 943 |
| #12 | (LSTM):ti,ab,kw OR (Generative Adversarial Network):ti,ab,kw OR (YOLO):ti,ab,kw OR (MobileNet):ti,ab,kw OR (ResNet):ti,ab,kw | 192 |
| #13 | (AlexNet):ti,ab,kw OR (VGGNet):ti,ab,kw OR (GoogLeNet):ti,ab,kw OR (Unet):ti,ab,kw OR (Radiomics):ti,ab,kw | 914 |
| #14 | (Radiomic):ti,ab,kw | 363 |
| #15 | #5 or #6 or #7 or #8 or #9 or #10 or #11 or #12 or #13 or #14 | 90678 |
| #16 | #4 and #15 | 400 |

3.Embase

| Search number | Query | Results |
| --- | --- | --- |
| #1 | 'crohn disease'/exp | 134098 |
| #2 | 'crohn disease':ab,ti OR 'crohns disease':ab,ti OR 'crohns enteritis':ab,ti OR 'terminal ileitis':ab,ti OR 'morbus crohn':ab,ti OR ileocolitis:ab,ti | 9447 |
| #3 | #1 OR #2 | 135413 |
| #4 | 'machine learning'/exp | 662636 |
| #5 | 'machine learning':ab,ti OR 'artificial intelligence':ab,ti OR 'transfer learning':ab,ti OR 'prediction model':ab,ti OR 'risk model':ab,ti OR 'risk score':ab,ti OR 'deep learning':ab,ti OR 'deep networks':ab,ti OR 'deep network':ab,ti OR 'ensemble learning':ab,ti OR 'random forest':ab,ti OR 'neural network':ab,ti OR 'neural networks':ab,ti OR 'k-nearest neighbor':ab,ti OR 'support vector machine':ab,ti OR svm:ab,ti OR 'gradient boosting machine':ab,ti OR nomogram:ab,ti OR xgboost:ab,ti OR adaboost:ab,ti OR lightgbm:ab,ti OR catboost:ab,ti OR 'gradient boosting':ab,ti OR 'decision tree':ab,ti OR 'regression trees':ab,ti OR 'naive bayesian':ab,ti OR 'multilayer perceptron':ab,ti OR 'bayesian network':ab,ti OR cnn:ab,ti OR rnn:ab,ti OR lstm:ab,ti OR 'generative adversarial network':ab,ti OR yolo:ab,ti OR mobilenet:ab,ti OR resnet:ab,ti OR alexnet:ab,ti OR vggnet:ab,ti OR googlenet:ab,ti OR unet:ab,ti OR radiomics:ab,ti OR radiomic:ab,ti | 624909 |
| #6 | #4 OR #5 | 930051 |
| #7 | #3 AND #6 | 2257 |

4.Web of science

| Search number | Query | Results |
| --- | --- | --- |
| #1 | TS=("Crohn Disease") OR TS=("Crohn's Disease") OR TS=("Crohns Disease") OR TS=("Crohn's Enteritis") OR TS=("Terminal Ileitis") OR TS=("Morbus Crohn") OR TS=("Ileocolitis") | 92014 |
| #2 | "Machine Learning " (Topic) OR "artificial intelligence" (Topic) OR "Transfer Learning" (Topic) OR "prediction model" (Topic) OR "risk model" (Topic) OR "risk score" (Topic) OR "deep learning" (Topic) OR "deep networks" (Topic) OR "deep network" (Topic) OR "Ensemble Learning" (Topic) OR "random forest" (Topic) OR "neural network" (Topic) OR "neural networks" (Topic) OR "K-Nearest Neighbor" (Topic) OR "Support vector machine" (Topic) OR "SVM" (Topic) OR "Gradient Boosting Machine" (Topic) OR "Nomogram" (Topic) OR "XGBoost" (Topic) OR "Adaboost" (Topic) OR "LightGBM" (Topic) OR "CatBoost" (Topic) OR "Gradient Boosting" (Topic) OR "Decision tree" (Topic) OR "Regression Trees" (Topic) OR "Naive Bayesian" (Topic) OR "Multilayer perceptron" (Topic) OR "Bayesian network" (Topic) OR "CNN" (Topic) OR "RNN" (Topic) OR "LSTM" (Topic) OR "Generative Adversarial Network" (Topic) OR "YOLO" (Topic) OR "MobileNet" (Topic) OR "ResNet" (Topic) OR "AlexNet" (Topic) OR "VGGNet" (Topic) OR "GoogLeNet" (Topic) OR "Unet" (Topic) OR "Radiomics" (Topic) OR "Radiomic" (Topic) | 2009068 |
| #3 | #1 AND #2 | 1237 |

**Supplementary Table S2** Summary of Study Characteristics

| No | First Author | Publication Year | Country | Study Design | Patient Source | Medication | Treatment Regimen | Definition of Treatment Response | Predicted Outcome | Follow-Up Time | Number of Predicted Event Cases | Total Sample Size | Number of Predicted Event Cases in the Training Set | Total Sample Size in the Training Set | Validation Approach | Overfitting Control Methods | Number of Predicted Event Cases in the Validation Set | Sample Size in the Validation Set | Missing Value Imputation Strategy | Feature selection strategies | Model Category | Features |
| --- | --- | --- | --- | --- | --- | --- | --- | --- | --- | --- | --- | --- | --- | --- | --- | --- | --- | --- | --- | --- | --- | --- |
| 1 | Pianpian Xia | 2025 | China | Retrospective Cohort Study | Single-Center | Adalimumab |  | Clinical remission was defined as a CD Activity Index (CDAI) ＜ 150. | Treatment Response | 52W | 12week model:86  48week model:149 | 244 | 12week model：69  48 week model: 119 | 12week model：195  48 week model: 195 | Random Split Validation | K-Fold Cross-Validation | 12 week model： 17  48 week model: 30 | 49 | Algorithmic interpolation | Sequential Framework Integrating Univariate Pre-Filtering with Multivariate Refinement | RF(random forest), GBM(gradient boosting machine), XGBoost(extreme gradient boosting), GBM(gradient boosting machine), CatBoost(categorical boosting, AdaBoost(adaptive boosting) | Clinical Features |
| 2 | Yiting Wang | 2025 | China | Retrospective Cohort Study | Single-Center | Infliximab | Patients received infliximab 5 mg/kg via intravenous infusion at baseline (Week 0), Week 2, and Week 6, followed by maintenance infusions every 8 weeks. | Anti-drug Antibody(ADA) Positivity if their ADA titer was ≥10 AU/mL 。 | Anti-drug Antibody Positivity | 42W | 145 | 606 | 116 | 485 | Random Split Validation | K-Fold Cross-Validation | 29 | 121 | Not Mentioned | Multivariate Analysis | RF(random forest), KNN(k-nearest neighbors), XGBoost(extreme gradient boosting), GBM(gradient boosting machine), LR(logistic regression), SVM(support vector machine), DT(decision tree) | Clinical Features |
| 3 | Y. Wang | 2025 | China | Retrospective Cohort Study | Single-Center | Anti-TNF therapy |  | Primary nonresponse to anti-TNF-α drugs was defined as failure to respond to induction regimen of anti-TNF agent, determined at 8–12 weeks after therapy initiation. Secondary loss of response was defined as CD-related surgery, switching medication, or unplanned hospital readmissions due to inflammatory activity. | Treatment Response | 52W | 47 | 105 | 35 | 79 | Random Split Validation | K-Fold Cross-Validation | 12 | 26 | Not Mentioned | Machine Learning-Driven Feature Selection | ViT(vision transformer) | Radiomic Features |
| 4 | Fang Wang | 2025 | China | Retrospective Cohort Study | Single-Center | Adalimumab | Patients received subcutaneous adalimumab at a dose of 160 mg (Week 0), 80 mg (Week 2), and 40 mg (Week 4), followed by a maintenance dose of 40 mg every other week. | The severity of disease activity was assessed according to the simpliffed Crohn’s Disease Activity Index score (Harvey Bradshaw Index, HBI)40 within the week before the first treatment and after 12 weeks of treatment. Clinical response was described as a decrease in HBI score of ≥ 3 points affer treatment. | Treatment Response | 12W | 38 | 68 | 38 | 68 | Bootstrap | Bootstrap | 38 | 68 | Not Mentioned | Multivariate Analysis | LR(logistic regression) | Clinical Features |
| 5 | Yun Qiu | 2025 | China | Retrospective Cohort Study | Multicenter | Infliximab | Crohn’s disease patients receiving ≥4 scheduled infliximab infusions (at weeks 0, 2, 6, and 14) were evaluated. | Clinical disease activity in patients was determined using the Crohn’s Disease Activity Index (CDAI). Clinical remission was defined as a CDAI of < 150 | Clinical Relapse | 104W | 235 | 746 | 26 week model:20, 52 week model:55, 144 week model:90 | 265 | Internal Random Split & External Validation。 | External validation | Internal validity：26 week model:8, 52week model:23, 144 week model:39；External validity：SAH 26 week model:2, SAH 52week model:51, SAH 144 week model:53；SYF 26 week model:26, SYF 52week model:34, SYF 144 week model:53 | Internal validity：114, External validity：SAH：176，SYF：191 | Median Imputation | Multivariate Analysis | XGBoost(extreme gradient boosting), LR(logistic regression), LASSO(Least Absolute Shrinkage and Selection Operator) | Clinical Features |
| 6 | Jingjing Liu | 2025 | China | Retrospective Cohort Study | Single-Center | Infliximab |  | Faecal calprotectin(FC )< 250 µg/g or ≥ 50% reduction from baseline at 26 ± 4 weeks post-Infliximab. | Treatment Response | 26W | 48 | 85 | 33 | 59 | Random Split Validation | Bootstrap | 15 | 26 | Multiple Imputation | Sequential Framework Integrating Univariate Pre-Filtering with Multivariate Refinement | LR(logistic regression) | Clinical Features |
| 7 | Xi Li | 2025 | China | Retrospective Cohort Study | Single-Center | Infliximab | Scheduled infliximab infusions (5–10 mg/kg) were administered intravenously at baseline (Week 0), Week 2, and Week 6, followed by maintenance therapy every 8 weeks | The definition included initiating alternative biological agents, requiring immunosuppressants or corticosteroids, infliximab dose escalation, and CD-related surgery. Additional criteria included a CD Activity Index(CDAI)> 150 or a reduction of less than 70% from baseline and/or evidence of mucosal recurrence, defined as a reduction in the Simple Endoscopic Score for CD (SES-CD) of < 50% or an SES-CD ≥ 3. | Treatment Response | 54W | 54 | 161 | 38 | 112 | Random Split Validation | k-Fold Cross-Validation&LASSO Regression | 16 | 49 | Not Mentioned | Multivariate Analysis | LR(logistic regression) | Combined Radiomic and Clinical Features |
| 8 | Shuai Li | 2025 | China | Retrospective Cohort Study | Multicenter | Infliximab | Standardized infliximab therapy (5–10 mg/kg IV) was administered to all CD patients: induction at weeks 0, 2, and 6, followed by maintenance every 8 weeks. | Initial response was defined as a decrease in the Harvey-Bradshaw Index (HBI) score of more than 2 points at week 12. The secondary non-response was defined as an initial response followed by a relapse or progression of CD. The therapeutic effect of IFX was evaluated by two gastroenterologists using an independent blind method based on the patients' HBI score, radiological and endoscopic examination results. If there was a discrepancy in opinions, the final decision was made by another senior physician. | Treatment Response | 54W | 69 | 220 | 47 | 156 | Internal Random Split & External Validation。 | Cross-Validation &Data Splitting | 22 | 64 | Deleted | Sequential Framework Integrating Univariate Pre-Filtering with Multivariate Refinement | LR(logistic regression) | Combined Radiomic and Clinical Features |
| 9 | Lixuan Li | 2025 | China | Retrospective Cohort Study | Single-Center | Infliximab | Patients were required to have completed ≥6 consecutive infliximab infusions. | The primary outcome was mucosal healing in CD patients on infliximab by week 30, defined as the absence of erosive mucosal lesions on colonoscopy. The binary presence/absence of erosive lesions was used in line with previous reports. The secondary outcome was defined as the Crohn’s Disease Activity Index (CDAI)＜150. | Post-Treatment Mucosal Healing | 30W | 86 | 179 | 60 | 125 | Random Split Validation |  | 26 | 54 | Deleted | Sequential Framework Integrating Univariate Pre-Filtering with Multivariate Refinement | LR(logistic regression) | Clinical Features |
| 10 | Zhuoyan Chen | 2025 | China | Retrospective Cohort Study | Multicenter | Infliximab | The therapeutic schedule involved intravenous infliximab 5 mg/kg administered at weeks 0, 2, and 6, with subsequent doses repeated at 8-week intervals. | Primary nonresponse was defined as the absence of response by week 14, while secondary loss of response was defined as the loss of response after an initial positive response within 52 weeks | Treatment Response | 52W | 99 | 134 | 62 | 84 | External validation | Data Splitting&Calibration Curves | 37 | 50 | Deleted | Sequential Framework Integrating Univariate Pre-Filtering with Multivariate Refinement | XGBoost(extreme gradient boosting), AdaBoost(adaptive boosting), KNN(k-nearest neighbors), NB(Naive Bayes), RF(random forest), BR(bridge regression), SVM(support vector machine) | Radiomic Features |
| 11 | Tian Yang | 2024 | China | Retrospective Cohort Study | Single-Center | Infliximab | Following induction, the maintenance regimen consisted of intravenous infliximab at a dose of 5–10 mg/kg administered every 8 weeks. | The outcome was defined by a multidisciplinary specialists group as a loss of responsiveness during week 14 to week 54 after therapy initiation, involving total Harvey Bradshaw indices (HBI) > 5 or an increase in the HBI ≥ 3 points, dose escalation, interval shortening, need for immunosuppressive therapy, corticosteroids or surgery related to CD, switching to other biological agents, and/or mucosal recurrence (a reduction from baseline in simple endoscopic score for CD (SES-CD) < 50% or SES-CD ≥ 3). | Treatment Response | 54W | 70 | 184 | 54 | 137 | Random Split Validation |  | 16 | 47 | Deleted | Sequential Framework Integrating Univariate Pre-Filtering with Multivariate Refinement | LR(logistic regression) | Combined Radiomic and Clinical Features |
| 12 | Yangdi Wang | 2024 | China | Retrospective Cohort Study | Multicenter | Infliximab | Standardized infliximab induction (5 mg/kg at weeks 0, 2, 6) was administered, with a clinical evaluation scheduled at Week 14. | The outcome needed to conform to the decrease of 50% in Simple Endoscopic Score for CD (SES-CD) relative to the baseline as recommended in expert consensus and prior reported clinical trials . | Post-Treatment Mucosal Healing | 14W | 69 | 231 | 33 | 112 | Internal Random Split & External Validation。 | External validation | Internal validity：14；External validity：22 | Internal validity：48；External validity：71 | Deleted | Sequential Framework Integrating Univariate Pre-Filtering with Multivariate Refinement | SVM(support vector machine) | Radiomic Features |
| 13 | Chao-Tao Tang | 2024 | China | Retrospective Cohort Study | Multicenter | Infliximab | All patients completed four scheduled infliximab infusions (5 mg/kg); response was evaluated after the fourth dose. | Assessed the clinical response according to the Patient-Reported Outcome 2(PRO2) score and adopted the standard of reducing the CD Activity Index(CDAI) by ＞100 for a small number of patients. Clinical remission in adult patients with CD was deﬁned as a Patient-Reported Outcome 2(PRO2) abdominal pain score or a Harvey-Bradshaw index, which are midterm goals. Endoscopic mucosal healing in patients with CD was deﬁned as a simpliﬁed endoscopic score for CD(SES-CD)≤3. | Treatment Response | 14W | 105 | 175 | 39 | 67 | Internal Random Split & External Validation。 | Bootstrap | Internal validity：26；External validity：40 | Internal validity：46；External validity：62 | Multiple Imputation Utilizing Actively Obtained Follow-Up Data | Sequential Framework Integrating Univariate Pre-Filtering with Multivariate Refinement | LR(logistic regression) | Combined Radiomic and Clinical Features |
| 14 | Fulong Song | 2024 | China | Retrospective Cohort Study | Single-Center | Infliximab | Patients received intravenous infliximab at a dose of 5–10 mg/kg at baseline (Week 0), Week 2, and Week 6, followed by maintenance infusions every 8 weeks. | The primary outcome was infliximab treatment failure within 1 year, which was defned as composite endpoints including primary non-response and secondary loss of response to infiximab. Primary non-response was defned as non-response to infiximab treatment during induction therapy (0–14 weeks), and secondary loss of response was defned as loss of clinical response (15–54 weeks) after primary response to infiximab treatment | Treatment Response | 52W | 52 | 137 | 37 | 95 | Random Split Validation | LASSO Regression | 15 | 42 | Deleted | Sequential Framework Integrating Univariate Pre-Filtering with Multivariate Refinement | LR(logistic regression) | Combined Radiomic and Clinical Features |
| 15 | Liang Chen | 2024 | China | Prospective Cohort Study | Multicenter | Anti-TNF therapy | Patients were treated with either infliximab (5 mg/kg) at weeks 0, 2, 6, and 14 (n=158), or ADA (160 mg at week 0, 80 mg at week 2, then 40 mg every 2 weeks; n=121). Serum trough levels were measured just before the scheduled week 14 dose. | The primary endoscopic response was defined as a decrease in the simple endoscopic score for CD(SES-CD) score by >50% compared with the baseline. | Post-Treatment Mucosal Healing | 14W |  | 443 | Infliximab：70； Adalimumab：66 | Infliximab：158，Adalimumab：121 | External validation | External validation&Cross-Validation |  | Infliximab：79，Adalimumab：58 | Deleted | Sequential Framework Integrating Univariate Pre-Filtering with Multivariate Refinement | LR(logistic regression) | Clinical Features |
| 16 | Weimin Cai | 2024 | China | Retrospective Cohort Study | Multicenter | Infliximab |  | The primary outcome focused on infliximab treatment failure within one year, defined as composite endpoints that encompass both primary non-response and secondary loss of response . Primary non-response refers to a lack of reaction to infliximab during the induction phase (0–14 weeks), while secondary loss of response is identified as a decline in clinical response (15–54 weeks) after initially responding to IFX treatment. The outcome was assessed by an experienced multidisciplinary team at our institution, which based its determinations on clinical symptoms, laboratory indices, radiological findings, and endoscopic results. | Treatment Response | 54W | 135 | 263 | 91 | 166 | External validation | External validation&LASSO Regression | 44 | 97 | Deleted | Sequential Framework Integrating Univariate Pre-Filtering with Multivariate Refinement | SVM(support vector machine) | Combined Radiomic and Clinical Features |
| 17 | Chao Zhu | 2023 | China | Retrospective Cohort Study | Multicenter | Infliximab | The therapeutic schedule involved intravenous infliximab 5 mg/kg administered at weeks 0, 2, and 6, with subsequent doses repeated at 8-week intervals. | The primary response was defined as a Harvey Bradshaw Index (HBI) reduction of more than 2 points at week 14. The clinical remission was defined as HBI <5 at week 54 . The secondary loss of response was evaluated comprehensively by two gastroenterologists for patients who had a primary response at week 14 and were no clinical remission at week 54, requiring alternative biologics, the addition of an immunomodulator, dose-escalation, or surgery. | Treatment Response | 54W | 64 | 181 | 37 | 102 | Internal Random Split & External Validation。 | External validation&LASSO Regression | Internal validity：16；External validity：11 | Internal validity：45；External validity：34 | Not Mentioned | Sequential Framework Integrating Univariate Pre-Filtering with Multivariate Refinement | LR(logistic regression) | Combined Radiomic and Clinical Features |
| 18 | Chen Yueying | 2023 | China | Retrospective Cohort Study | Single-Center | Infliximab | All participants received intravenous infliximab at a dose of 5 mg/kg as per the standardized treatment protocol. | Clinical response to infliximab therapy was considered as a Harvey-Bradshaw Index (HBI) reduction of > 2 points from baseline . The failure of infliximab was divided into primary non-response and secondary loss of response. Primary non-response was defined as a lack of clinical response (decrease in HBI ≥ 3 points) at week 14, whereas secondary loss of response was defined as a relapse after an initial response to IFX within 52 weeks requiring alternative treatments , HBI > 5 or an increase in the HBI ≥ 3 points, and/or mucosal infammation (a reduction from baseline in simple endoscopic score for CD (SES-CD) < 50% or SES-CD ≥ 3). | Treatment Response | 52W | 64 | 188 | 43 | 112 | Random Split Validation | LASSO Regression | 21 | 76 | Not Mentioned | Multivariate Analysis | LR(logistic regression) | Radiomic Features |
| 19 | Rosario Medina-Medina | 2023 | Spain | Prospective Cohort Study | Multicenter | Anti-TNF therapy | The study cohort was nearly equally distributed between infliximab (or biosimilar) therapy (50.4%) and adalimumab therapy (49.6%). | The criteria to consider：the absence of response were: CD Activity Index(CDAI)> 150; endoscopic or radiologic activity; purulent drainage after a gentle finger compression; new fistula; dose escalation; switch to a different drug; addition of other immunosuppressants; surgery or endoscopic dilation. | Treatment Response | 52W | 16 | 113 | 16 | 113 | Phased Validation |  |  |  | Not Mentioned | Sequential Framework Integrating Univariate Pre-Filtering with Multivariate Refinement | LR(logistic regression) | Clinical Features |
| 20 | Chao Zhu | 2022 | China | Retrospective Cohort Study | Multicenter | Infliximab | Infliximab was administered intravenously at a dose of 5 mg/kg at weeks 0, 2, and 6, and subsequently every 8 weeks thereafter. | Mucosal Healing(MH) was defined as an simple endoscopic scorefor CD(SES-CD)< 3 with the disappearance of mucosal ulcers at the 26-week colonoscopy in CD patients who had inflammatory intestinal segments with ulcers at the baseline colonoscopy. | Post-Treatment Mucosal Healing | 26W | 37 | 106 | 25 | 73 | Random Split Validation |  | 12 | 33 | Deleted | Sequential Framework Integrating Univariate Pre-Filtering with Multivariate Refinement | LR(logistic regression) | Combined Radiomic and Clinical Features |
| 21 | Xuehua Li | 2022 | China | Retrospective Cohort Study | Multicenter | Infliximab | Standard infliximab induction (5 mg/kg at weeks 0, 2, 6) was administered, with evaluation performed at Week 14. | The coutcome needed to conform to the decrease of 50% in simple endoscopic scorefor CD(SES-CD) relative to the baseline as recommended in expert consensus and prior reported clinical trials . Two gastroenterologists retrospectively evaluated the SES-CD through the report description and endoscopic pictures.The endoscopic images were retrospectively evaluated by two gastroenterologists | Post-Treatment Mucosal Healing | 14W | 73 | 244 | 35 | 119 | Internal Random Split & External Validation。 | LASSO Regression | Internal validity：15；External validity：23 | Internal validity：51；External validity：74 | Not Mentioned | Sequential Framework Integrating Univariate Pre-Filtering with Multivariate Refinement | LR(logistic regression) | Combined Radiomic and Clinical Features |
| 22 | Pavine L.C. Lefevre | 2022 | United States | Secondary Analysis of RCT Data | Multicenter | Certolizumab Pegol | Patients in PRECISE 1 received Certolizumab Pegol 400 mg subcutaneously (weeks 0, 2, 4) plus maintenance every 4 weeks, or placebo. In PRECISE 2, patients received open-label induction (400 mg at weeks 0, 2, 4); Week 6 responders were randomized to CZP 400 mg or placebo every 4 weeks. Follow-up extended to Week 26 in both studies. | Crohn’s Disease Activity Index ≤150 and fecal calprotectin concentration(FC) ≤250 μg/g at Weeks 6 or 26. | Treatment Response | 26W | 6 week model：466，26 week model:38，External validity：not mentioned | 964 | 6 week model：466, 26 week model:38 | 6 week model：636, 26 week model:427 | Internal Random Split & External Validation。 | Bootstrap |  | 328 | Not Mentioned | Sequential Framework Integrating Univariate Pre-Filtering with Multivariate Refinement | LR(logistic regression) | Clinical Features |
| 23 | Jing Feng | 2022 | China | Retrospective Cohort Study | Single-Center | Infliximab |  | The secondary loss of response is deﬁned in those patients who initially respond to inﬂiximab therapy and subsequently lost clinical response. The CD Activity Index(CDAI) value was used as a reference standard to determine whether patients responded to inﬂiximab. The cut-oﬀ values were set at a CDAI ≤150 for inactive disease and >150 for active disease to indicate whether or not a patient showed a clinical response after treatment with inﬂiximab. In brief, CDAI over 150 indicates loss of response. Patients with dose intensiﬁcation or replacement with another anti-TNF drugs were also included as the secondary loss of response in this study | Treatment Response | 54W | 157 | 322 | 122 | 257 | Random Split Validation | Data Splitting | 35 | 65 | Not Mentioned | Sequential Framework Integrating Univariate Pre-Filtering with Multivariate Refinement | LR(logistic regression) | Combined Radiomic and Clinical Features |
| 24 | Chen Yueying | 2021 | China | Retrospective Cohort Study | Single-Center | Infliximab | Enrolled patients, all naïve to anti-TNF therapy, received intravenous infliximab (Janssen) at a dose of 5 mg/kg. Doses were administered at weeks 0, 2, and 6, with subsequent maintenance infusions every 8 weeks. | The primary outcome was the secondary loss of response assessed by a multidisciplinary team of experienced experts as a relapse or deterioration after an initial clinical response (a reduction of ≥2 points in the Harvey-Bradshaw Index (HBI)) during week 54, based on the following approach: (1) elevated clinical disease activity index (HBI＞5) and inflammatory marker (C-reactive protein＞8.0 mg/L) ; (2) indicated need for therapy modiffcation, including dose escalation, alternative biological agents, corticosteroids, combinations of immunosuppressive therapy, or Crohn’s disease-related surgery . | Treatment Response | 54W | 69 | 136 |  |  | Ten-fold cross-validation | K-Fold Cross-Validation |  |  | Not Mentioned | Sequential Framework Integrating Univariate Pre-Filtering with Multivariate Refinement | LR(logistic regression) | Clinical Features |
| 25 | Xiao-Qi Ye | 2021 | China | Retrospective Cohort Study | Multicenter | Infliximab | Centers administered infliximab induction (5 mg/kg at weeks 0, 2, 6). Patients were excluded based on predefined criteria. | The primary outcome was defined as a decrease in Crohn's Disease Endoscopic Index of Severity (CDEIS) of < 50% from baseline at week 14 after the first infliximab infusion. On the contrary, response was defined as a decrease in CDEIS of 50% from baseline at week 14. The secondary outcome was mucosal healing, defined as CDEIS < 1.5 at week 14. Prior surgery was defined as resection of a part of the gut, strictureplasty for stenosing complications, or a fistulectomy/fistulotomy for complicated perianal disease. | Post-Treatment Mucosal Healing | 14W | 88 | 343 | 62 | 240 | Random Split Validation | Bootstrap | 26 | 103 | Deleted | Sequential Framework Integrating Univariate Pre-Filtering with Multivariate Refinement | LR(logistic regression) | Clinical Features |
| 26 | Yong Li | 2021 | China | Retrospective Cohort Study | Single-Center | Infliximab | All eligible patients received infliximab intravenous administration of 5 mg/kg at weeks 0, 2 and 6 respectively in the induction therapy, and before which they were pre-medicated with methylprednisolone 1 mg/kg, to reduce the risk of adverse reactions during the infusion. | Clinical outcome was primarily evaluated as clinical response, defined as a decrease in CD Activity Index (CDAI) of 70 points or more from the baseline (week 0) by the end of the induction period (week 14) and no gastrointestinal surgery performed | Treatment Response | 14W | 51 | 174 | 36 | 122 | Random Split Validation+10-fold cross validation | Bootstrap | 15 | 52 | Not Mentioned | Sequential Framework Integrating Univariate Pre-Filtering with Multivariate Refinement | RF(random forest) | Clinical Features |
| 27 | Li Li | 2021 | China | Prospective Cohort Study | Single-Center | Infliximab | Induction therapy was initiated with intravenous infliximab administered at a dose of 5 mg/kg at weeks 0, 2, and 6. | Primary nonresponse of IFX was defined as a failure of the CD Activity Index (CDAI) to drop more than 70 points or to <150 at week 14 after IFX administration. Situations in which patients received an alternative therapy schedule such as an escalation of corticosteroid therapy, switching to other agents or having the surgery before week 14 were also defined as Primary nonresponse. | Treatment Response | 14W | 57 | 260 | 45 | 182 | Bootstrap | Bootstrap |  |  | Deleted | Sequential Framework Integrating Univariate Pre-Filtering with Multivariate Refinement | RF(random forest) | Clinical Features |
| 28 | Yueying Chen | 2021 | China | Retrospective Cohort Study | Single-Center | Infliximab | Infliximab therapy was initiated, with maintenance doses of 5–10 mg/kg every 8 weeks. | A Crohn’s disease activity index (CDAI) reduction of more than 70 points or a decrease of Crohn’s Disease Activity Index (CDAI) of more than 2 points from baseline was considered a clinical response to infliximab therapy. Secondary loss of response was defined by a multi-disciplinary team of experienced experts at our institution during week 54, including a relapse of CD after an initial response, such as CDAI was rising again to the baseline level or even higher, and the requirement of an alternative biological agent, corticosteroid, or surgery. | Treatment Response | 54W | 43 | 186 |  |  | Ten-fold cross validation | K-Fold Cross-Validation&LASSO Regression |  |  | Deleted | Multivariate Analysis | LR(logistic regression) | Radiomic Features |
| 29 | Thomas Billiet | 2015 | Beligum | Retrospective Cohort Study | Single-Center | Infliximab | The therapeutic protocol involved an induction phase with infliximab (5 mg/kg), with doses scheduled at weeks 0, 2, and 6. | Primary nonresponse was defined at Week 14 for CD patients with luminal disease as complete absence of clinical improvement after induction therapy, and as no reduction of at least 50% of the number of draining fistulas for CD patients with perianal fistulising disease, based on physician global assessment | Treatment Response | 14W | 16 | 201 |  |  | Phased Validation |  |  |  | Deleted | Sequential Framework Integrating Univariate Pre-Filtering with Multivariate Refinement | LR(logistic regression) | Clinical Features |

**Supplementary Table S3** Meta-Analysis of C-indices for Internal and External Validation of Clinical Feature-Based Machine Learning Models Predicting Anti-TNF-α Treatment Response in Crohn's Disease

| Clinical featrues | Internal Validation Set | | | External Validation Set | | |
| --- | --- | --- | --- | --- | --- | --- |
|  | n | c-index(95%CI) | Tau2 | n | c-index(95%CI) | Tau2 |
| Model |  |  |  |  |  |  |
| XGBoost | 1 | 0.752 (0.598-0.945) | NA | 1 | 0.654 (0.557-0.768) | NA |
| RF | 2 | 0.923 (0.877-0.972) | 0 |  |  |  |
| LR | 11 | 0.740 (0.674-0.813) | 0.014 | 3 | 0.775 (0.622-0.966) | 0.028 |
| SVM |  |  |  | 1 | 0.710 (0.620-0.813) | NA |
| Medication |  |  |  |  |  |  |
| Adalimumab | 2 | 0.912 (0.852-0.975) | 0.001 |  |  |  |
| Infliximab | 10 | 0.725 (0.657-0.800) | 0.011 | 4 | 0.733 (0.672-0.800) | 0.023 |
| Certolizumab | 1 | 0.710 (0.634-0.795) | NA | 1 | 0.710 (0.634-0.795) | NA |
| Anti-TNF-α therapy | 1 | 0.919 (0.863-0.978) | NA |  |  |  |
| Response |  |  |  |  |  |  |
| Primary Non-Response | 8 | 0.822(0.745-0.907) | 0.014 | 2 | 0.820(0.606-1.00） | 0.041 |
| Secondary Loss of response | 5 | 0.697(0.627-0.776 | 0 | 2 | 0.657(0.572-0.756) | 0 |
| Treatment Failure | 1 | 0.657(0.495-0.872) | NA | 1 | 0.710(0.620-0.813) | NA |
| Overall | 14 | 0.773 (0.711-0.841) | 0.016 | 5 | 0.733 (0.645-0.833) | 0.014 |

Note: Treatment failure encompasses primary non-response (PNR) and secondary loss of response (SLOR).
Abbreviations: XGBoost: extreme gradient boosting; RF: random forest; LR: logistic regression; SVM: support vector machine.

**Supplementary Table S4** Meta-Analysis of C-indices for Internal and External Validation of Radiomics-Based Machine Learning Models Predicting Anti-TNF-α Treatment Response in Crohn's Disease

| Radiomic Features | Internal Validation Set | | | External Validation Set | | |
| --- | --- | --- | --- | --- | --- | --- |
|  | n | c-index(95%CI) | Tau2 | n | c-index(95%CI) | Tau2 |
| Model |  |  |  |  |  |  |
| XGBoost |  |  |  | 1 | 0.910 (0.847-0.977) | NA |
| ViT | 1 | 0.745 (0.590-0.941) | NA |  |  |  |
| LR | 7 | 0.849 (0.788-0.915) | 0.005 | 2 | 0.904 (0.848-0.965) | 0 |
| SVM |  |  |  | 1 | 0.720 (0.630-0.823) | NA |
| Medication |  |  |  |  |  |  |
| Infliximab | 7 | 0.849 (0.788-0.915) | 0.005 | 4 | 0.865 (0.783-0.955) | 0.008 |
| Anti-TNF therapy | 1 | 0.745 (0.590-0.941) | NA |  |  |  |
| Response |  |  |  |  |  |  |
| Primary Non-Response | 1 | 0.954(0.900-1.00) | NA | 1 | 0.902(0.833-0.977) | NA |
| Secondary Loss of response | 4 | 0.806(0.742-0.875) | 0 | 1 | 0.909(0.815-1.00) | NA |
| Treatment Failure | 3 | 0.839(0.766-0.918) | 0.001 | 2 | 0.815(0.648-1.00) | 0.024 |
| Overall | 8 | 0.840 (0.781-0.904) | 0.005 | 4 | 0.865 (0.783-0.955) | 0.008 |

Note: Treatment failure encompasses primary non-response (PNR) and secondary loss of response (SLOR).
Abbreviations: XGBoost: extreme gradient boosting; ViT: vision transformer; LR: logistic regression; SVM: support vector machine.

**Supplementary Table S5** Meta-Analysis of C-indices for Internal and External Validation of Machine Learning Models Integrating Clinical and Radiomic Features to Predict Anti-TNF-α Treatment Response in Crohn's Disease

| Combined Radiomic and Clinical Features | Internal Validation Set | | | External Validation Set | | | |  |
| --- | --- | --- | --- | --- | --- | --- | --- | --- |
|  | n | c-index(95%CI) | Tau2 | | n | c-index(95%CI) | Tau2 | |
| Model |  |  |  | |  |  |  | |
| LR | 6 | 0.830 (0.780-0.883) | 0 | | 1 | 0.921 (0.835-1.000) | NA | |
| SVM |  |  |  | | 1 | 0.850 (0.778-0.929) | NA | |
| Medication |  |  |  | |  |  |  | |
| Infliximab | 6 | 0.830 (0.780-0.883) | 0 | | 2 | 0.883 (0.816-0.955) | 0.001 | |
| Response |  |  |  | |  |  |  | |
| Secondary Loss of response | 5 | 0.830(0.778-0.885) | 0 | | 1 | 0.921(0.836-1.00) | NA | |
| Treatment Failure | 1 | 0.832(0.678-1.00) | NA | | 1 | 0.850(0.778-0.929) | NA | |
| Overall | 6 | 0.830 (0.780-0.883) | 0 | | 2 | 0.883 (0.816-0.955) | 0.001 | |

Note: Treatment failure encompasses primary non-response (PNR) and secondary loss of response (SLOR).
Abbreviations: LR: logistic regression; SVM: support vector machine.

**Supplementary Figure S1** Forest Plot of Meta-Analysis Results for C-Indices of Different Clinical Feature-Based Machine Learning Models in Training Sets Predicting Response to Anti-TNF Therapy

**Supplementary** **Figure S2** Forest Plot of Meta-Analysis Results for C-Indices of Different Clinical Feature-Based Machine Learning Models in Validation Sets Predicting Response to Anti-TNF Therapy

**Supplementary** **Figure S3** Forest Plot of Meta-Analysis Results for C-Indices of Different Clinical Feature-Based Machine Learning Models in Internal Validation Sets Predicting Response to Anti-TNF Therapy

**Supplementary** **Figure S4** Forest Plot of Meta-Analysis Results for C-Indices of Different Clinical Feature-Based Machine Learning Models in External Validation Sets Predicting Response to Anti-TNF Therapy

**Supplementary** **Figure S5** Forest Plot of Meta-Analysis Results for C-Indices of Different Radiomic Feature-Based Machine Learning Models in Training Sets Predicting Response to Anti-TNF Therapy

**Supplementary** **Figure S6** Forest Plot of Meta-Analysis Results for C-Indices of Different Radiomic Feature-Based Machine Learning Models in Validation Sets Predicting Response to Anti-TNF Therapy

**Supplementary** **Figure S7** Forest Plot of Meta-Analysis Results for C-Indices of Different Radiomic Feature-Based Machine Learning Models in Internal Validation Sets Predicting Response to Anti-TNF Therapy

**Supplementary** **Figure S8** Forest Plot of Meta-Analysis Results for C-Indices of Different Radiomic Feature-Based Machine Learning Models in External Validation Sets Predicting Response to Anti-TNF Therapy

**Supplementary** **Figure S9** Forest Plot of Meta-Analysis Results for C-Indices of Different Combined Clinical and Radiomic Feature-Based Machine Learning Models in Training Sets Predicting Response to Anti-TNF Therapy

**Supplementary** **Figure S10** Forest Plot of Meta-Analysis Results for C-Indices of Different Combined Clinical and Radiomic Feature-Based Machine Learning Models in Validation Sets Predicting Response to Anti-TNF Therapy

**Supplementary** **Figure S11** Forest Plot of Meta-Analysis Results for C-Indices of Different Combined Clinical and Radiomic Feature-Based Machine Learning Models in Internal Validation Sets Predicting Response to Anti-TNF Therapy

**Supplementary** **Figure S12** Forest Plot of Meta-Analysis Results for C-Indices of Different Combined Clinical and Radiomic Feature-Based Machine Learning Models in External Validation Sets Predicting Response to Anti-TNF Therapy

**Supplementary Figure S13** Funnel plot of machine learning-predicted treatment response in the training cohort.

**Supplementary Figure S14** Funnel plot of machine learning-predicted treatment response in the validation cohort.

**Supplementary Figure S15** Funnel plot of machine learning-predicted mucosal healing in the training cohort.

**Supplementary Figure S16** Funnel plot of machine learning-predicted mucosal healing in the validation cohort.

**Supplementary** **Figure S17** Forest Plot of Meta-Analysis Results for C-Indices of Machine Learning Models Based on Different Modeling Variables in Training Sets Predicting Mucosal Healing in Response to Anti-TNF Therapy

**Supplementary** **Figure S18** Forest Plot of Meta-Analysis Results for C-Indices of Machine Learning Models Based on Different Modeling Variables in Validation Sets Predicting Mucosal Healing in Response to Anti-TNF Therapy
